# Pathogenesis-related protein 10 in resistance to biotic stress: progress in elucidating functions, regulation and modes of action

**DOI:** 10.3389/fpls.2023.1193873

**Published:** 2023-07-04

**Authors:** Natasha dos Santos Lopes, Ariana Silva Santos, Diogo Pereira Silva de Novais, Carlos Priminho Pirovani, Fabienne Micheli

**Affiliations:** ^1^ Departamento de Ciências Biológicas (DCB), Centro de Biotecnologia e Genética (CBG), Universidade Estadual de Santa Cruz (UESC), Ilhéus-Bahia, Brazil; ^2^ Centre de Coopération Internationale en Recherche Agronomique pour le Développement (CIRAD), Unité Mixte de Recherche Amélioration Génétique et Adaptation des Plantes Meditérranéennes et Tropicales (UMR AGAP Institut), Montpellier, France

**Keywords:** defense mechanism, pathogens, systematic review, stress, signal transduction

## Abstract

**Introduction:**

The Family of pathogenesis-related proteins 10 (PR-10) is widely distributed in the plant kingdom. PR-10 are multifunctional proteins, constitutively expressed in all plant tissues, playing a role in growth and development or being induced in stress situations. Several studies have investigated the preponderant role of PR-10 in plant defense against biotic stresses; however, little is known about the mechanisms of action of these proteins. This is the first systematic review conducted to gather information on the subject and to reveal the possible mechanisms of action that PR-10 perform.

**Methods:**

Therefore, three databases were used for the article search: PubMed, Web of Science, and Scopus. To avoid bias, a protocol with inclusion and exclusion criteria was prepared. In total, 216 articles related to the proposed objective of this study were selected.

**Results:**

The participation of PR-10 was revealed in the plant’s defense against several stressor agents such as viruses, bacteria, fungi, oomycetes, nematodes and insects, and studies involving fungi and bacteria were predominant in the selected articles. Studies with combined techniques showed a compilation of relevant information about PR-10 in biotic stress that collaborate with the understanding of the mechanisms of action of these molecules. The up-regulation of PR-10 was predominant under different conditions of biotic stress, in addition to being more expressive in resistant varieties both at the transcriptional and translational level.

**Discussion:**

Biological models that have been proposed reveal an intrinsic network of molecular interactions involving the modes of action of PR-10. These include hormonal pathways, transcription factors, physical interactions with effector proteins or pattern recognition receptors and other molecules involved with the plant’s defense system.

**Conclusion:**

The molecular networks involving PR-10 reveal how the plant’s defense response is mediated, either to trigger susceptibility or, based on data systematized in this review, more frequently, to have plant resistance to the disease.

## 1 Introduction

In their natural environment, biotic and abiotic stresses often challenge plants. Viruses, bacteria, fungi, oomycetes, nematodes and insects are the main biotic factors that seriously impact the plant growth, survival and productivity, threatening global food security (Nejat and Mantri, 2017; Alonso et al., 2019; VanWallendael et al., 2019). To survive these and other stresses, plants have developed an immune response involving a series of complex molecular mechanisms activating a cascade of genes that encode pattern recognition receptors (PRRs), effectors, and signaling and defense molecules (Nejat and Mantri, 2017). Among the molecules recruited to defend the plant against stress, several studies highlighted the pathogenesis-related proteins (PRs).

PRs are small proteins that perform various biochemical functions in the cell, from promoting growth and development to defending against biotic and abiotic stresses (Breiteneder et al., 1989; Walter et al., 1990; Hashimoto et al., 2004; Liu and Ekramoddoullah, 2006; van Loon et al., 2006; Jiang et al., 2015; Castro et al., 2016; Kattupalli et al., 2021). Currently, PRs are classified into 17 families (PR-1 to PR-17) that include chitinases, glucanases, thaumatins, defensins, peroxidases, endoproteinases, thionins, lipid transfer proteins (LTPs) and ribonucleases (van Loon et al., 2006; Sels et al., 2008). The multigenic family of PR-10 consists of small acidic proteins, predominant in the cytosol of cells, with some exceptions found in the nucleus, cell membrane or forming complexes with other proteins in the plant cells’ apoplast and mitochondria (Ekramoddoullah, 2004; Choi et al., 2012; Lee et al., 2012; Fan et al., 2015; Ma et al., 2018). Some functions have already been reported for PR-10, such as RNase and DNase activity; antimicrobial and antifungal action; and binding to various ligands such as cytokinin, flavonoids, abscisic acid, melatonin and brassinosteroids, suggesting a role in plant hormone regulation (Pasternak et al., 2006; Xie et al., 2010; Fernandes et al., 2013; Wang et al., 2014; Fan et al., 2015; Ma et al., 2018; Sliwiak et al., 2018). In addition, PR-10 proteins control the synthesis of flavonoids through binding to intermediate metabolites, they transport lipids (Casañal et al., 2013), have norcoclaurine synthase (Lee and Facchini, 2010) and aldo/keto reductase enzymatic activity (Jain and Kumar, 2015).

The PR-10 proteins’ action against biotic stress has been discussed; some review articles have sought to compile data regarding their structure, function and biochemical aspects (Fernandes et al., 2013; Jain and Kumar, 2015; Sinha et al., 2020). However, little is known about the molecular mechanisms played by PR-10 in biotic stress in plants. New questions that seek more in-depth answers on this aspect are necessary to close these gaps and the systematization of data can help with this. One widely used method in compiling experimental data to respond to complex answers is the systematic review (SR). The SR aims to provide an assemblage of relevant and up-to-date research information, which shows the state of the art on a given topic, using well-defined methods and guidelines to avoid bias (Needleman, 2002; Moher et al., 2009). The SR is commonly applied in health areas, for example, in the investigation of medication side effects, to assist in medical decision making (Swingler et al., 2003; Hunter et al., 2020). However, SR has gained space in the biological and agricultural sciences areas to elucidate molecular mechanisms, pathosystems and host responses to various types of stress, as shown in reviews on pathogen interaction with plant crops (da Silva et al., 2021; Soares et al., 2021; Santos et al., 2023), as well as on reducing the abiotic factor effects (Santos et al., 2018; dos Santos et al., 2022; Oliveira et al., 2022; Nascimento et al., 2023).

Much knowledge about PR-10 roles in biotic stress has been accumulated in literature databases. However, it is presented as loose pieces of a puzzle, particularly regarding the PR-10 action mechanism. Therefore, this SR aims to elucidate the mechanism of action of PR-10 against biotic stresses, based on systematizing the literature published between the years 2003-2021.

## 2 Materials and methods

The SR was conducted using the software StArt (State of the Art through Systematic Review) version Beta 3.4, developed by the Research Laboratory in Software Engineering (LAPES) of the Federal University of São Carlos (UFSCar) (Available at: http://lapes.dc.ufscar.br/tools/start_tool). The review followed the PRISMA (Preferred Reporting Items for Systematic Reviews and Meta-Analyses) guidelines (Page et al., 2021), which consist of three steps: planning, execution and summarization.

### 2.1 Planning

For the planning stage, a search protocol (available in the Supplementary material at https://www.frontiersin.org/articles/10.3389/fpls.2023.1193873/full#supplementary-material) was elaborated, containing important information for developing the SR, such as: objective, search strings, inclusion and exclusion criteria, definition of study types, extraction strategy, data summarization and research questions (**Table 1**). The questions that guided the SR (**Table 1**) were based on the Population Intervention Comparison Results (PICOS) strategy (da Costa Santos et al., 2007), which directs what the research question needs to specify, avoiding biased answers (**Table 2**) (Wright et al., 2007).

**Table 1:** Guiding questions for this SR.

Research questions	
1- What were the plant species in which PR-10 was characterized? 2- What biotic stressors are reported in PR-10 studies? 3- Are the methodologies in studies with PR-10 effective to elucidate the mechanisms of action against biotic stresses? 4- Is there differential expression of PR-10 in varieties susceptible or resistant to biotic stresses? 5- What are the functions of PR-10 in defense against biotic stresses? 6- What are the mechanisms of action that PR-10 performs?

**Table 2:** Description of the PICOS strategy used to formulate the SR research questions.

	Description	Question components
P	Population	PR-10 in biotic stress in plants
I	Intervention	Elucidation of behavior, function and modes of action of PR-10 in biotic stress
C	Comparison	Plants submitted to a given type of biotic stress compared to non-submitted plants or resistant plants to biotic stress compared to susceptible plants
O	Outcome	Uncover the role of PR-10 in resistance against biotic stresses in plants
S	Type of study	Scientific articles

### 2.2 Execution

To answer the research questions (**Table 1**), articles were searched in three databases, PubMed, Scopus, and Web of Science, using a single search string (PR10 OR “PR 10” OR PR-10) AND (“biotic stress” OR “biotic stresses”). Boolean connectors “OR” and “AND” were used in the string to group synonymous keywords and main terms. The selected files were imported in BIBITEX and MEDILINE format into the StArt software (vs. Beta 3.4) where the automated selection was made based on reading the titles and abstracts, using the inclusion and exclusion criteria established in the protocol as a reference (available in the Supplementary material at https://www.frontiersin.org/articles/10.3389/fpls.2023.1193873/full#supplementary-material), thus characterizing articles as accepted or rejected and excluding duplicates. Articles that were accepted based on one or more inclusion criteria were read completely to extract information that answered the SR questions. During the full reading phase, it was possible to exclude some studies based on at least one of the exclusion criteria.

### 2.3 Summarization

At this stage, the information extracted from the studies was summarized in graphs, tables and figures. Bibliometric analyzes were performed based on the selected articles’ metadata using the bibliometrix package (Aria and Cuccurullo, 2017) of the *R* statistical environment (R.C. Team, 2021) in the following manner: (i) identifying journals that contributed with the largest number of studies included in the review; (ii) generating collaboration networks between authors, institutions and countries; (iii) and generating co-occurrence network of terms in the works’ titles. Vosviewer software (van Eck and Waltman, 2014) was used for graphical visualization of collaboration and co-occurrence networks. To reduce any bias in preparing the SR, the PRISMA checklist was used (available in the Supplementary material at https://www.frontiersin.org/articles/10.3389/fpls.2023.1193873/full#supplementary-material).

## 3 Results

### 3.1 Selection of studies and bibliometric indicators

**Figure 1:**
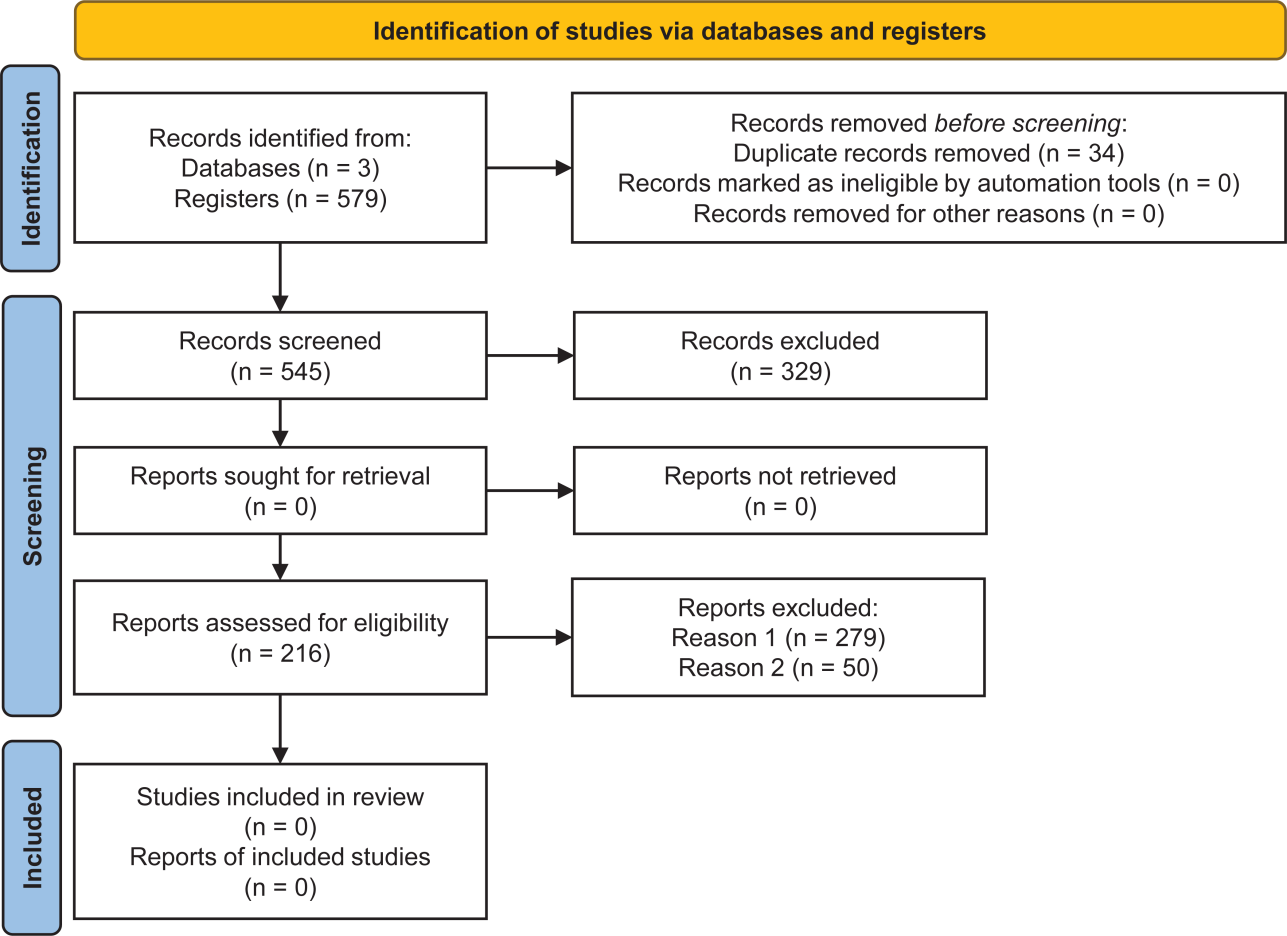
Flowchart with identification and selection of studies according to PRISMA guidelines (Page et al., 2021).

A total of 579 articles were returned from searches in the databases, with 518, 28 and 33 from the databases PubMed, Web of Science, and Scopus, respectively. Electronic searches in each database corresponded to 89.46%, 4.83% and 5.69% of studies in the aforementioned databases. Although the same string was used in different databases, PubMed contributed to the SR with more studies. In the first selection of articles in the StArt software, 329 articles were excluded because they were ineligible for the review’s inclusion criteria, with 279 being excluded based on reading the titles and abstracts and 50 based on reading the complete articles. The automatic tool detected 34 duplicate articles. Therefore, a resulting sample of 216 articles, which met at least one inclusion criterion, was included in the SR (**Figure 1**). To ensure there was no bias in this SR, the PRISMA checklist was completed (available in the Supplementary material at https://www.frontiersin.org/articles/10.3389/fpls.2023.1193873/full#supplementary-material), confirming the transparency and quality of the preparation and execution of the process steps.

**Figure 2:**
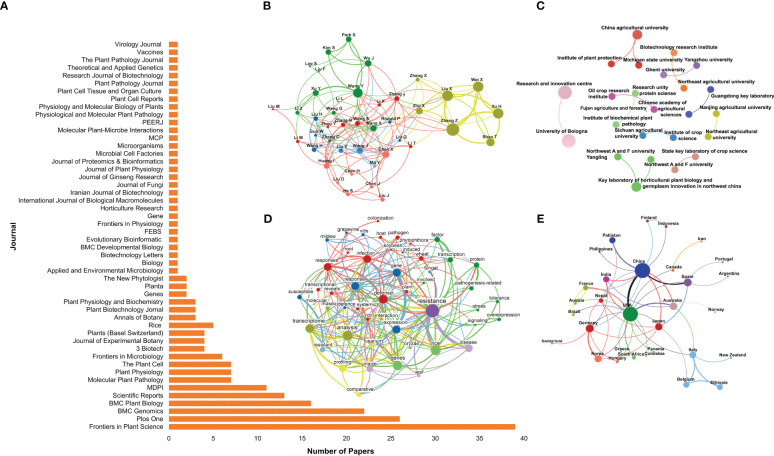
Bibliometric indicators of scientific knowledge production and dissemination on the SR subject of the selected articles. **(A)** Article distribution by journal. **(B)** Collaboration network between authors. **(C)** Collaboration network between research institutions. **(D)** Network of co-occurrence of words in the selected articles’ titles. **(E)** Collaboration network between countries. In **(B, C, E)**, the size of the circle represents the number of article occurrences (the larger the circle, the greater the article number), the thickness of the lines represent the number of collaborations between authors, and research institutions or countries (the thicker the line, the greater the number), respectively. Colors define node clusters that have similar patterns of collaboration. In **(D)**, the circle’s size represents the number of articles with occurrences of the term in the title (the larger the circle, the greater the occurrence). The line thickness defines the number of co-occurrences of two terms (the thicker the line, the greater the co-occurrence). Colors define clusters of terms that frequently appear together in article titles.

The bibliometric indicators allowed identifying the scientific journals that published the most about the role of PR-10 in biotic stress (**Figure 2A**). From the 216 eligible articles, the journals *Frontiers in Plant Science*, *PLOS One*, *BMC Genomics*, *BMC Plant Biology* and *Scientific Reports* – all with an impact factor greater than 3.0 – contributed with the higher number of publications. The journals listed in **Figure 2A** are subjected to peer review and have a reliability index. Collaboration networks among leading authors, institutions and countries conducting research about the role of PR-10 in biotic stresses were generated based on eligible articles. The collaborative profile of the scientists who research the most on the subject formed five clusters represented by the green, yellow, red, blue, and pink colors (**Figure 2B**). The yellow cluster was connected to all the other groups; it contained authors, such as Liu X., Zhang Z., Wei X., and Xu H., who highly contributed to the subject of this SR (large size hubs; **Figure 2B**). In contrast, the pink, red, and blue groups, even interacting together, contained only a few hubs, inferring the low contribution to the SR of the articles published by the authors included in these groups (**Figure 2B**). The metadata of the research and teaching institutions that most produced and collaborated in studies on the role of PR-10 in biotic stress are represented in **Figure 2C**. Eleven clusters were formed and highlighted in different colors. The institutions of each cluster collaborated with other institutions from the same cluster, but did not form connection with institutions from other clusters. The institutions that collaborated with the highest level of publications are highlighted by the size of the hub, such as the China Agricultural University, Research and Innovation Center, and Key Laboratory of Horticulture in red, lilac, and light green clusters, respectively (**Figure 2C**). Keywords such as “resistance,” “response” and “infection” showed a higher occurrence in the selected articles related to the role of PR-10 in biotic stress (**Figure 2D**). China, USA, Japan, Spain, Germany, Australia, and India, in the decreasing order of the hub size, stood out in terms of countries that most produce and disseminate scientific knowledge regarding the role of PR-10 in biotic stress (**Figure 2E**).

**Figure 3:**
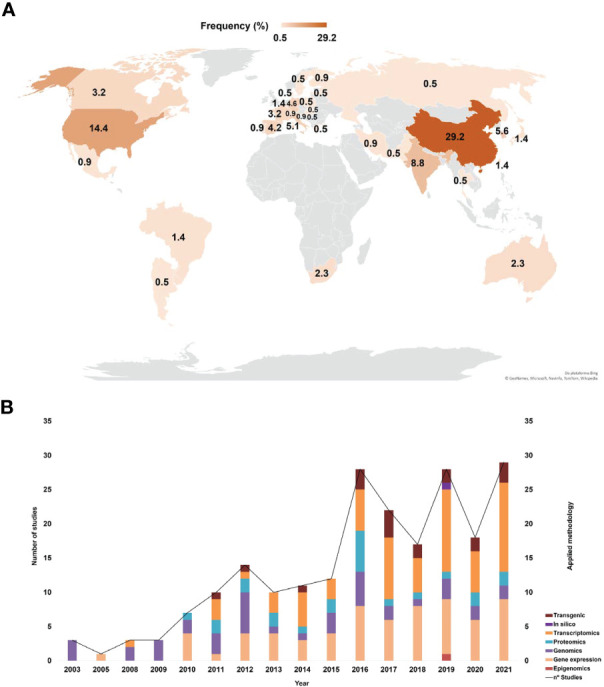
Bibliometric indicators of the selected articles. **(A)** Main countries that published about PR-10. **(B)** Main methods applied in the selected articles related to PR-10.

The articles included in this SR represented all the continents (**Figure 3A**). Countries such as China, the USA and India stood out in terms of higher production with 29.2%, 14.4% and 8.8%, respectively. Brazil represented 1.4% of the eligible articles in this SR – these articles investigated mainly the effects of PR-10 on fungal cells and on omics (proteomics and transcriptomics) to elucidate the plant defense responses against hemibiotrophic fungi. The selected articles were published between 2003 and 2021 (**Figure 3B**). The methodological strategies used in these articles were categorized into seven main fields: transgenics, transcriptomics, proteomics, genomics, gene expression, epigenomics and *in silico* analyses. The analysis of the methodological strategies per publication year allowed observing that omics techniques are present in all studies, except in those published in 2005. The most used methodological strategies were gene expression and transcriptomics, while only few studies used only *in silico* analyses to characterize PR-10 (e.g., in 2019) (**Figure 3B**).

### 3.2 Functions played by PR-10 in biotic stress and cellular localization

Articles eligible for the SR investigated the role of PR-10 and its cellular location in plant species when subjected to biotic stress. **Table S1** shows the compilation of plant species and stressors. The species that predominated in the investigation of the role of PR-10 in biotic stress were *Oryza sativa* (20.37%), *Zea mays* (9.25%), *Vitis vinifera* (8.79%) and *Triticum aestivum* (7.87%). The identified stressors included fungi (65.74%), bacteria (24.53%), oomycetes (7.40%), insects (6.94%), nematodes (4.62%) and viruses (3.24%). In *Oryza sativa*, the most prominent interactions occurred with fungi (47.7%) and bacteria (40.9%); in *Zea mays* and *Triticum aestivum*, fungi were the most significant with 90% and 94.1%, respectively; whereas in *Vitis vinifera*, the highest occurrence was with fungi (63.15%) (**Table S1**).

From the 216 selected articles, only 3.24% investigated the subcellular localization of PR-10. From them, 2.3% found that the PR-10 location occurred in the cytoplasm/cytosol of the plant cell (Choi et al., 2012; Lee et al., 2012; Ma et al., 2018). However, other studies identified the PR-10 presence in other cell compartments, such as cell membrane (0.46%) (Fan et al., 2015), nucleus (0.46%) (Lee et al., 2012) and mitochondria (0.46%) (Ma et al., 2018) or in the apoplast (0.46%) co-located with other proteins such as VDCA and LRR1 (Choi et al., 2012). In addition, a grape PR-10 was characterized as containing a mitochondrial targeting peptide (**Table S1**) (Katam et al., 2015).

The data systematization revealed several functions PR-10 performs in biotic stress. About 65% of the articles – which mainly used transcriptomics and proteomics as methodological strategies – indicated that PR-10 proteins were involved in defense, which could be considered its main role. However, other studies have shown more specific functions along with the defense role, such as participation in cell death, hypersensitivity response (HR) and systemic acquired resistance (SAR) signaling over long distances, in addition to RNase and DNase activity, antifungal and antimicrobial action, storage and transport of ligands, callose deposition, allergenicity, signaling in hormonal pathways (salicylic acid/SA, jasmonic acid/JA and abscisic acid/ABA), synthesis of secondary metabolites, symbiosis marker gene and induction of oxidative stress (**Table S1**).

### 3.3 Differential levels of PR-10 expression under biotic stress

**Figure 4:**
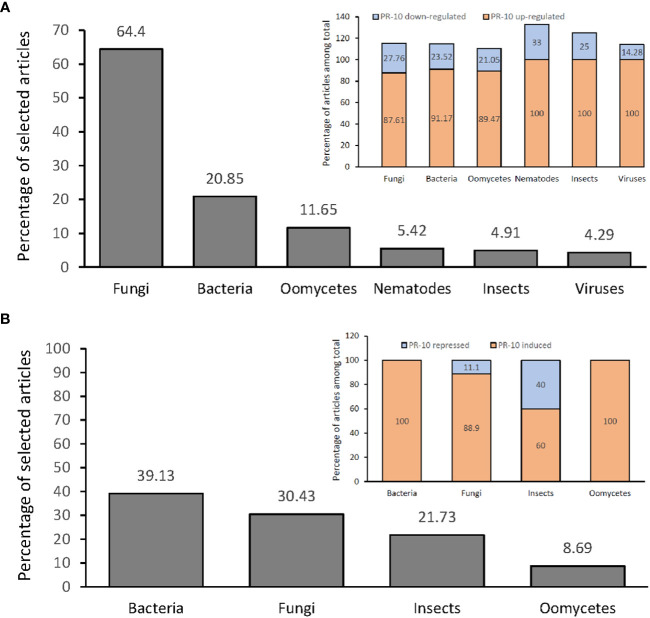
Percentage of articles showing PR-10 expression (transcriptional level) or accumulation (translational level) in plants subjected to biotic stresses. **(A)** Percentage total of articles (gray graph) showing up- or down- regulation of PR-10 expression in plants subjected to different stressors (inset). **(B)** Percentage total of articles (gray graph) showing induction or repression of PR-10 proteins in plants subjected to different stressors (inset). Some articles contained data about both up- and down- regulation, or both induction and repression of PR-10, for this reason the sum of some percentages does not add up to 100%.

From the articles selected in this SR, an investigation of the differential expression profile of PR-10 was carried out at the transcriptional (**Figure 4A**) and translational levels (**Figure 4B**). In both, there was a predominance of up-regulation or induction of transcripts and proteins, respectively, that codify PR-10, for all evaluated stressor treatments. Notably, some of the articles identified, in the same study, the up- and down-regulation (transcripts) or induction and repression (protein) of PR-10 at the different times tested. Thus, the sum of some percentages presented in the SR does not add up to 100%.

Studies that measured PR-10 transcription in plants submitted to fungal stress reached 64.4% of the selected articles, from which 87.61% and 24.76% corresponded to studies in which PR-10 genes were up-regulated and down-regulated, respectively (**Figure 4A**). About 20.85% of the articles corresponded to PR-10 transcription in plants infected by bacteria; PR-10 genes were up-regulated and down-regulated in 91.17% and 23.52% of the articles, respectively (**Figure 4A**). Regarding plants stressed by oomycetes, the percentage of articles was 11.65%, with 89.47% and 21.05% of them corresponding to studies in which PR-10 genes were up-regulated and down-regulated, respectively (**Figure 4A**). About 5.52% of the articles corresponded to PR-10 transcription in plants infected by nematode; PR-10 genes were up-regulated and down-regulated in 100% and 33.33% of the articles, respectively (**Figure 4A**). Regarding plants stressed by insects, the percentage of articles was 4.91%, with 100% and 25% of them corresponding to studies in which PR-10 genes were up-regulated and down-regulated, respectively (**Figure 4A**). Finally, regarding plants stressed by viruses, the percentage of articles was 4.29%, with 100% and 14.28% of them corresponding to studies in which PR-10 genes were up-regulated and down-regulated, respectively (**Figure 4A**). From these studies, 10.42% evaluated PR-10 expression with more than one pathogen per study (**Figure 4A**). The results indicated that fungi and bacteria stood out as the main stressor agents in studies of differential expression of transcripts in plants under stress and that PR-10 genes were up-regulated in a greater proportion than down-regulated in response to all the evaluated stresses.

The transcriptional profile of PR-10 in susceptible or resistant plant varieties subjected to different biotic stresses was addressed in 51.28% of the articles selected in this SR. In 58.75% of these articles, PR-10 genes were up-regulated in the resistant variety. In a total of 52.51% of the studies, PR-10 genes were up-regulated in the susceptible variety, often at later times and in smaller amounts compared to the resistant one (**Table S2**). Other studies evaluated the differential levels of transcription of PR-10 in plants inoculated by the pathogen compared to the non-inoculated ones (control), having a total of 34.35% articles investigating this. Regarding inoculated versus control plants, 93.64% of the articles showed up-regulated PR-10 transcripts and 25% down-regulated ones (**Table S2**).

The repression of PR-10 transcription was represented in 23.71% of the articles, included those related to i) plants under the action of endophytic agents and mycorrhizae, which form mutualistic symbiotic associations with plants; ii) the evaluation of more than one PR-10 in different genotypes or plant tissues; and iii) some resistant plant varieties. In other cases, the down-regulation of PR-10 occurred shortly after it was up-regulated, showing a positive expression profile in the early stages of infection and then a decline (**Table S2**).

Among the 216 articles selected in this RS, 10.64% evaluated the accumulation of PR-10 at the protein level in plants subjected to different stresses. From this total, 39.13% investigated plants inoculated with bacteria, and in 100% of these articles, PR-10 proteins were induced. A percentage of 30.43% of the articles evaluated the protein accumulation in response to fungi; in 88.9% of these articles, PR-10 proteins were induced and in 11.1%, they were repressed. The articles that evaluated the accumulation of PR-10 at the protein level in plants subjected to insects corresponded to 21.73% of them; 60% of them showed an induction of PR-10 and 40% a repression. The articles that evaluated the accumulation of PR-10 in plants subjected to the action of oomycetes, which totaled 8.69%; and 100% of them addressed induced PR-10 (**Figure 4B**; **Table S3**). At the protein level, the results showed that fungi and bacteria also stood out as main stressor agents in articles with PR-10 accumulation in plants subjected to biotic stress. However, in this case, unlike the panorama of studies with PR-10 transcripts, studies involving bacteria had a higher percentage than those involving fungi. High accumulation of PR-10 is predominant over low accumulation for all stressors studied. Furthermore, induction occurred in all resistant plant species subjected to stress caused by fungi, insects and oomycetes and in avirulent or incompatible interactions in bacteria. PR-10 was induced in *Capsicum annuum* when interacting with bacteria, but in insects, it decayed, causing its repression. Finally, repression was observed in varieties susceptible to fungal pathogens (**Figure 4B**; **Table S3**).

### 3.4 Molecules acting in regulation of PR-10 expression

**Figure 5:**
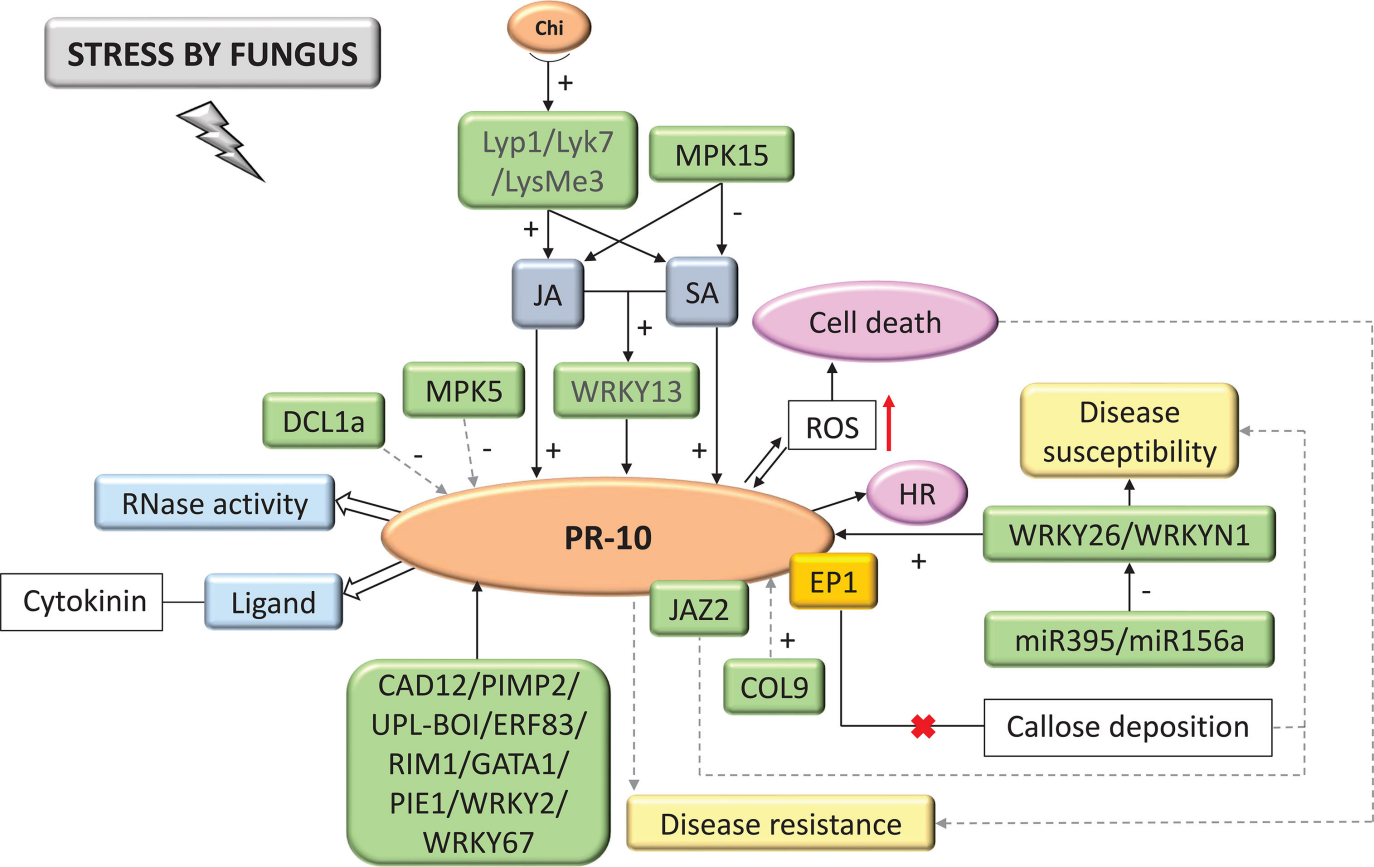
Biological model of a signaling network in which PR-10 is involved in the responses of plants subjected to fungal stress. Thin, non-dotted arrows indicate a direct PR-10 reaction. Dotted arrows indicate an indirect reaction, involving steps or molecules as yet unknown or not shown. Thick white arrows indicate the role PR-10 played (blue boxes). (+) or (-) indicates positive or negative regulation, and increase or decrease of the performed function, respectively. Red arrow indicates ROS increase. Green boxes represent molecules that regulate PR-10 expression or interact directly with it. The mustard-colored box indicates an effector. Gray boxes indicate signaling pathways involving phytohormones. Yellow-colored boxes indicate the acquired response. Open boxes indicate processes or molecules that involve the function performed by PR-10. Pink circles indicate reactions that contribute to disease resistance. The red (x) indicates the interruption of the action.

**Figure 6:**
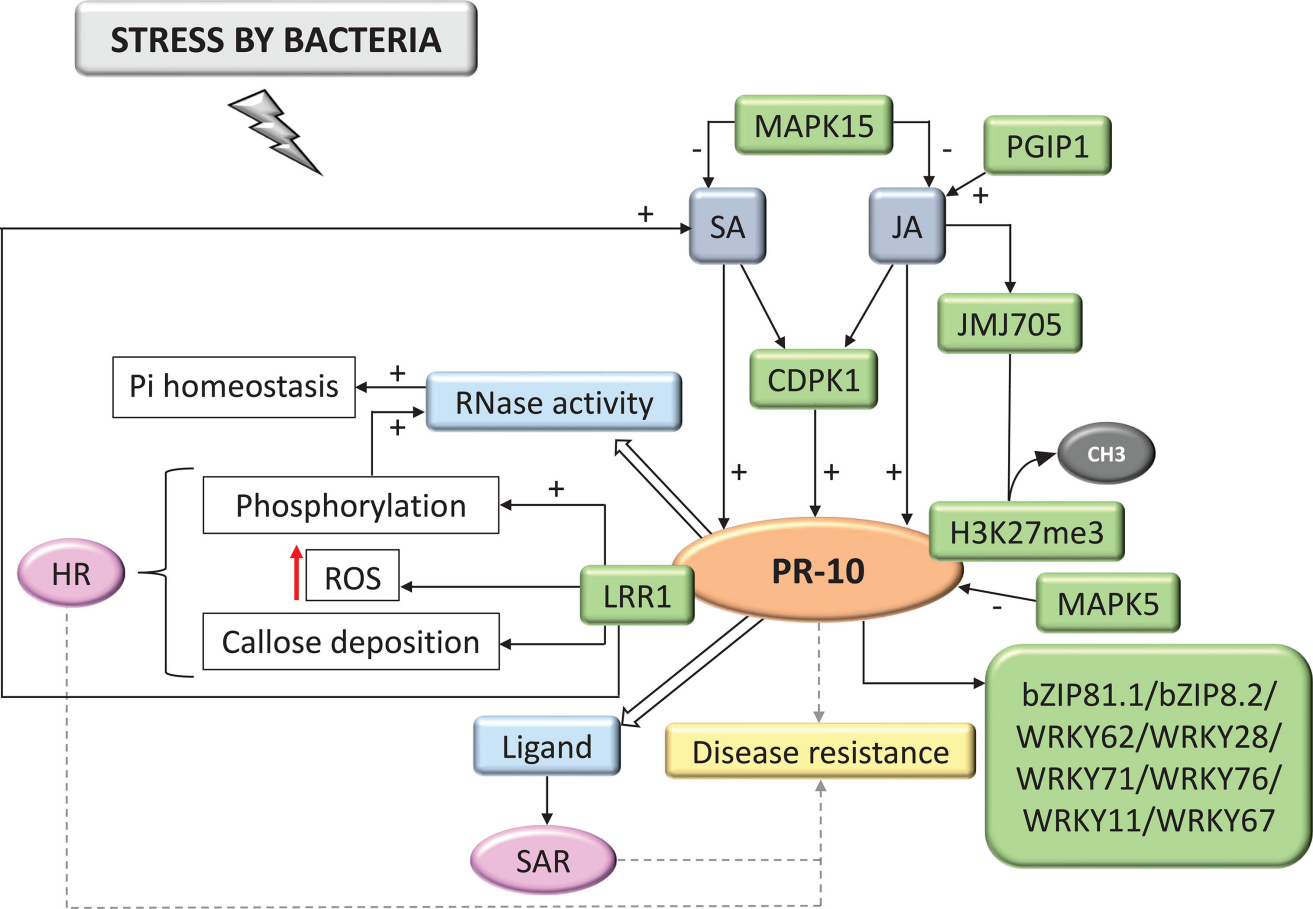
Biological model of a signaling network in which PR-10 is involved in the responses of plants subjected to stress caused by bacteria. Thin, nondotted arrows indicate a direct PR-10 reaction. Dotted arrows indicate an indirect reaction, involving steps or molecules as yet unknown or not shown. Thick white arrows indicate the role played by PR-10 (blue boxes). (+) or (-) indicate positive or negative regulation, and increase or decrease of the performed function, respectively. Red arrow indicates ROS increase. Green boxes represent molecules that regulate PR-10 expression or interact directly with it. Gray boxes indicate signaling pathways involving phytohormones. Yellow-colored box indicates the acquired response. Open boxes indicate processes or molecules that involve the function performed by PR-10. Pink circles indicate reactions that contribute to disease resistance. Black circle indicates methyl group release.

**Figure 7:**
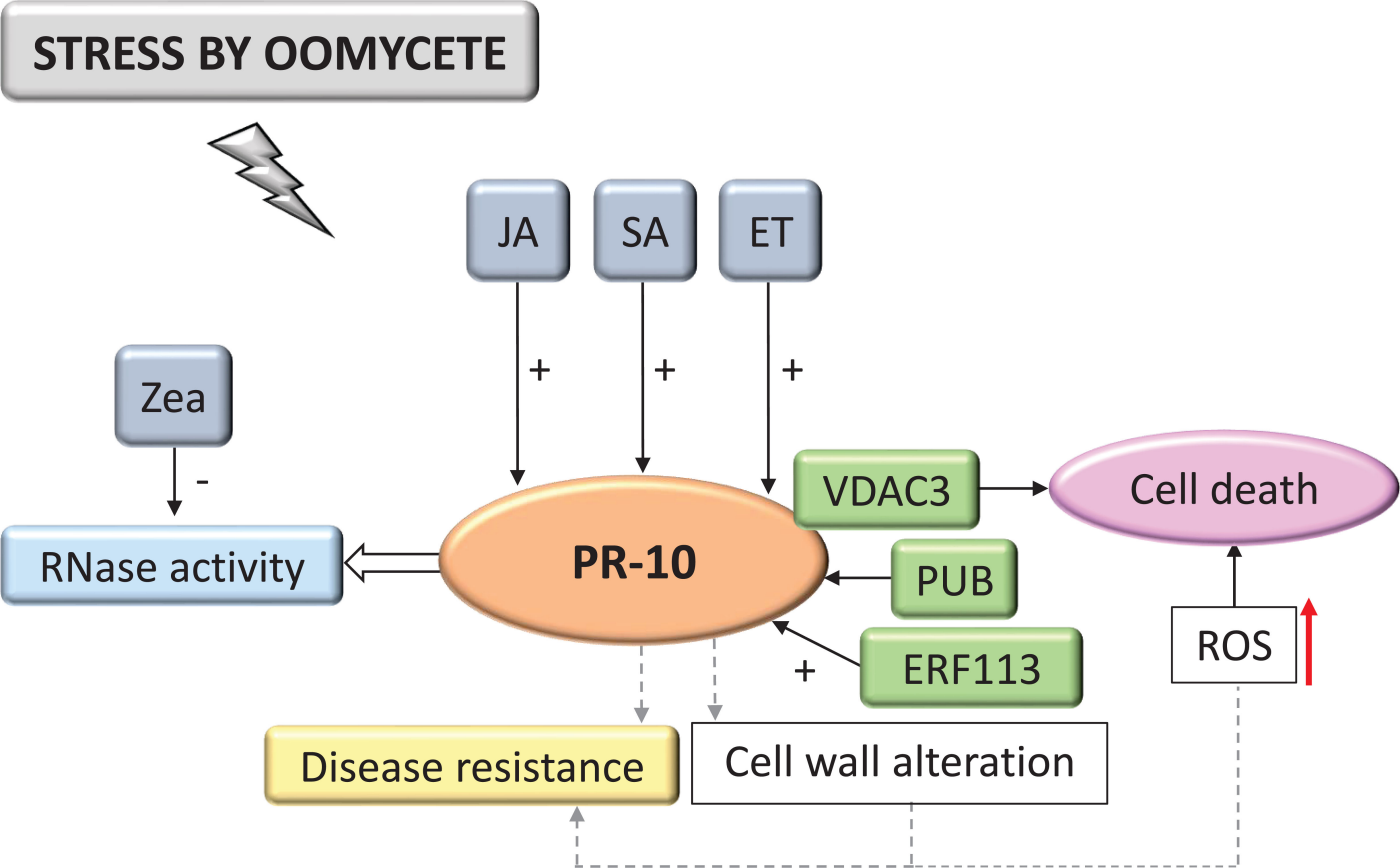
Biological model of a signaling network in which PR-10 is involved in the responses of plants subjected to stress caused by oomycetes. Thin, nondotted arrows indicate a direct PR-10 reaction. Dotted arrows indicate an indirect reaction, involving steps or molecules as yet unknown or not shown. Thick white arrow indicates the role played by PR-10 (blue box). (+) or (-) indicate positive or negative regulation, and increase or decrease of the performed function, respectively. Red arrow indicates ROS increase. Green boxes represent molecules that regulate PR-10 expression or interact directly with it. Gray boxes indicate signaling pathways involving phytohormones. Yellow-colored box indicates the acquired response. Open boxes indicate processes or molecules that involve the function performed by PR-10. Pink circle indicates reaction that contribute to disease resistance.

**Figure 8:**
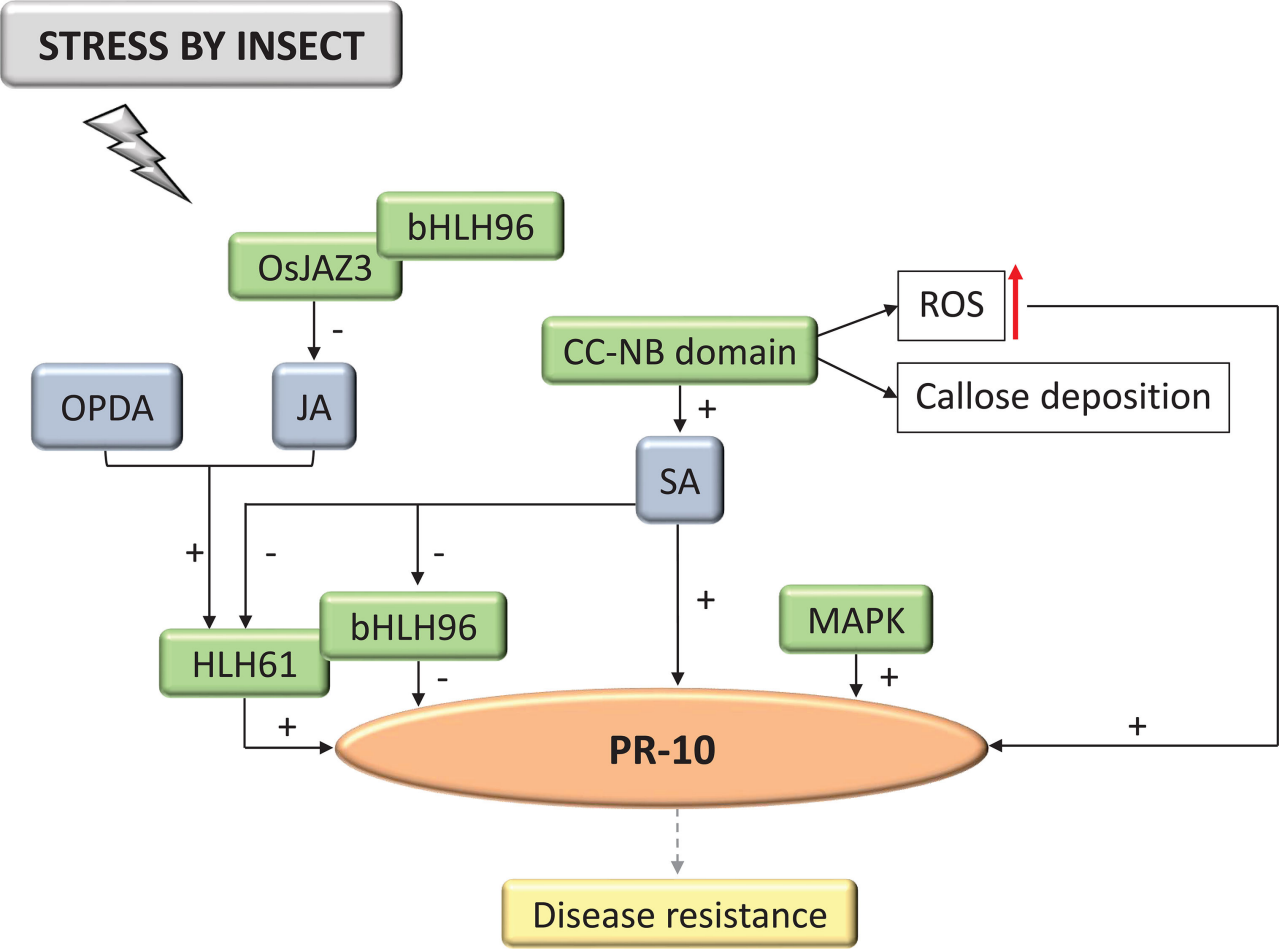
Biological model of a signaling network in which PR-10 is involved in the responses of plants subjected to stress caused by insects. Thin, non-dotted arrows indicate a direct PR-10 reaction. (+) or (-) indicate positive or negative regulation, and increase or decrease of the performed function, respectively. Red arrow indicates ROS increase. Green boxes represent molecules that regulate PR-10 expression or interact directly with it. Gray boxes indicate signaling pathways involving phytohormones. Yellow-colored box indicates the acquired response. Open boxes indicate processes or molecules that involve the function performed by PR-10.

**Figure 9:**
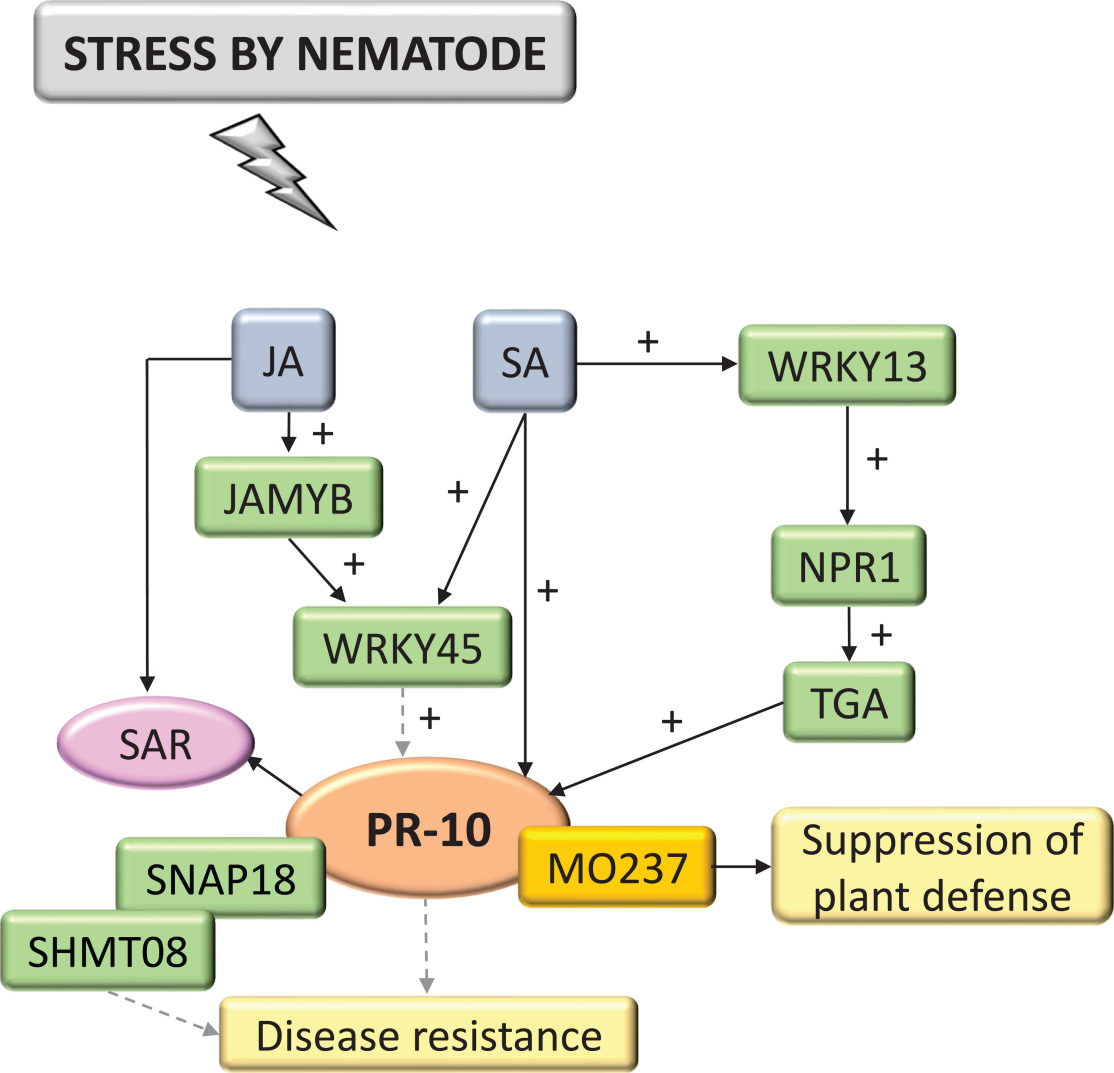
Biological model of a signaling network in which PR-10 is involved in the responses of plants subjected to stress caused by nematodes. Thin, non-dotted arrows indicate a direct PR-10 reaction. Dotted arrows indicate an indirect reaction, involving steps or molecules as yet unknown or not shown. (+) indicates positive regulation and increase of the performed function. Green boxes represent molecules that regulate PR-10 expression or interact directly with it. The mustard-colored box indicates an effector. Gray boxes indicate signaling pathways involving phytohormones. Yellow-colored boxes indicate the acquired response. Pink circle indicates reaction that contribute to disease resistance.

**Figure 10:**
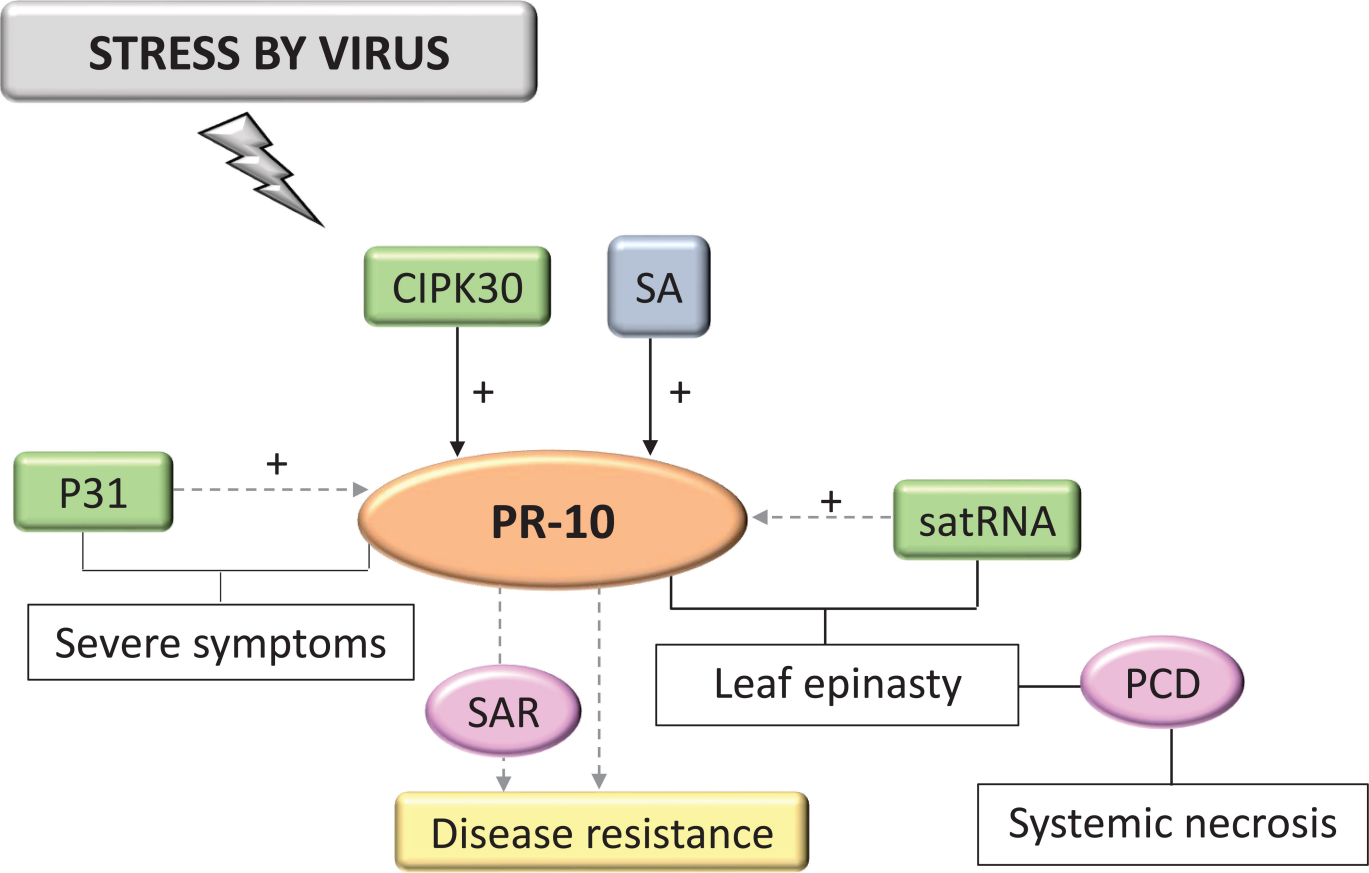
Biological model of a signaling network in which PR-10 is involved in the responses of plants subjected to stress caused by viruses. Thin, non-dotted arrows indicate a direct PR-10 reaction. Dotted arrows indicate an indirect reaction, involving steps or molecules as yet unknown or not shown. (+) indicates positive regulation. Green boxes represent molecules that regulate PR-10 expression. Gray box indicates signaling pathways involving phytohormones. Yellow-colored box indicates the acquired response. Pink circles indicate reactions that contribute to disease resistance.

The analysis of articles addressing the differential expression of transcripts in plants overexpressing or silencing transcription factors (TFs) and molecules involved in the defense response (**Table 3**) revealed that these molecules regulated negatively or positively the PR-10 expression after inoculating the plants with the stressor (**Figures 5**–**10**). These data pointed to molecules that acted on PR-10 regulation pathways and contributed to constructing the interaction network models proposed in this SR (**Figures 5**–**10**).

**Table 3:** Molecules directly or indirectly involved in regulating PR-10 in plants subjected to biotic stressors.

Plant species	Molecules that regulate PR-10 expression	Type	Reference
*Glycine max*	ERF113	Transcription factor	Zhao et al., 2017
*Gossypium hirsutum/Gossypium barbadense*	Lyp1/Lyk7/LysMe3	Lysin motif (LysM) containing protein pattern recognition receptors (PRRs)	Xu et al., 2017
*Lens culinaris*	UPL-BOI	E3 ubiquitin protein ligase	Khorramdelazad et al., 2018
*Malus domestica*	WRKY26/WRKYN1	Transcription factor	Zhang et al., 2017
*Medicago truncatula*	miR1510b*	MicroRNAs (miRNAs)	Devers et al., 2011
*Nicotiana benthamiana*	RNA4-encoded P31	P31 Protein	Wu et al., 2014
*Nicotiana tabacum*	WRKY2	Transcription factor	Dabi et al., 2020
*Oryza sativa*	WRKY	Transcription factor	Jimmy and Babu, 2019
*Oryza sativa*	ERF922	Transcription factor	Liu et al., 2012
*Oryza sativa*	ERF83	Transcription factor	Tezuka et al., 2019
*Oryza sativa*	MPK15	Mitogen-activated protein kinase	Hong et al., 2019
*Oryza sativa*	CIPK30	CBL (calcineurin B-like proteins)-interaction protein kinase protein	Liu et al., 2017
*Oryza sativa*	CDPK1	Calcium-dependent protein kinases	He et al., 2018
*Oryza sativa*	WRKYIIa (WRKY62, WRKY28, WRKY71 and WRKY76)	Transcription factor	Peng et al., 2010
*Oryza sativa*	WRKY67	Transcription factor	Vo et al., 2017
*Oryza sativa*	Dcl1a	Dicer protein	Salvador-Guirao et al., 2018
*Oryza sativa*	MAPK5	Mitogen-activated protein kinase	Xiong and Yang, 2003
*Oryza sativa*	CC and NB from BPH14 domain	Leucine-rich repeat protein	Hu et al., 2017
*Oryza sativa*	WRKY IIc (WRKY11)	Transcription factor	Lee et al., 2018
*Oryza sativa*	JMJ705	Histone lysine demethylase	Li et al., 2013
*Oryza sativa*	COL9	CONSTANS-like genes	Liu et al., 2016
*Oryza sativa*	bZIP81.2/bZIP81.1	Transcription factor	Liu et al., 2019
*Oryza sativa*	HLH61/bHLH96	Basic helix-loop-helix proteins	Wang et al., 2019
*Oryza sativa*	PGIP1	Polygalacturonase-inhibiting proteins	Wu et al., 2019
*Paeonia lactiflora*	WRKY13	Transcription factor	Wang et al., 2019
*Solanum lycopersicum*	ERFs and RAV2	Transcription factor	Li et al., 2011
*Triticum aestivum*	GATA1	Transcription factor	Liu et al., 2019
*Triticum aestivum*	PIE1	Transcription factor	Zhu et al., 2014
*Triticum aestivum*	PIMP2	Transcription factor	Wei et al., 2017
*Triticum aestivum*	CAD12	Cinnamyl alcohol dehydrogenase enzyme	Rong et al., 2016
*Triticum aestivum*	RIM1	Transcription factor	Shan et al., 2016
*Vitis vinifera*	MPK15	Mitogen-activated protein kinase	Hong et al., 2019
*Vitis vinifera*	PUB	Stress-responsive U-box protein	Jiao et al., 2017

Among the articles, 15.74% focused on investigating molecules related to PR-10 regulation. Several methodologies that included transgenics (silencing or overexpressing molecules), RT-qPCR (analyzing the differential profile of gene expression) and yeast two-hybrid assay (Y2H) or fluorescence complementation assay (BiFC) (to check the interaction between molecules) were used to unravel their roles in the positive or negative regulation of PR-10, which contributed to understanding the mechanism of action of PR-10. The TFs WRKY, AP2/ERF, bZIP, RAV2, GATA1 and MYB were predominant in these studies, demonstrating the diversity of pathways including PR-10 that can trigger a defense response.

PRRs that recognize the invader and act in defense against pathogen attack, as well as molecules involved in post-transcriptional and epigenetic regulation such as miR1510b*, Dcl1a and JMJ705, were also characterized for regulating PR-10. The MAPKs (protein kinases activated by mitogens) identified in this study, showed activity in the negative regulation of PR-10. Several other molecules that participate in plant defense responses in the interaction with various stressor agents were identified in the PR-10 regulation process (**Table 3**).

### 3.5 Biological models of PR-10 signaling in response to plant stressor agents

Among the selected articles in this SR, 35.18% allowed systematizing data, which together, infer about possible biological models of molecular signaling networks of plants subjected to biotic stresses involving PR-10.

#### 3.5.1 Fungi

A total of 65.74% of the studies evaluated the interaction between plants and fungi. Based on these studies, chitin and pathogen-associated molecular patterns (PAMP) are recognized by LysMe3, Lyk7 and Lyp1 PRR types that lead to activating defense processes increasing plant resistance (Xu et al., 2017). These processes include the SA and JA hormonal signaling pathways, activating the expression of PR-10, as well as the WRKY13 TF, which also acts by activating the transcription of this protein (**Figure 5**) (Wang et al., 2019). Other molecules were seen as active in the PR-10 signaling network, where they positively regulated PR-10 transcription, such as COL9 (Constans-Like9) (Liu et al., 2016); CAD12 (cinnamyl alcohol dehydrogenase enzyme) (Rong et al., 2016); UPL-BOI (E3 ubiquitin protein ligase) (Khorramdelazad et al., 2018); and the TFs WRKY2 (Dabi et al., 2020), WRKY67 (Vo et al., 2017), WRKY26, WRKYN1 (Zhang et al., 2017), PIMP2 (Wei et al., 2017), ERF83 (Tezuka et al., 2019), RIM1 (Shan et al., 2016), GATA1 (Liu et al., 2019), and PIE1 (Zhu et al., 2014). Systematized data showed that in stress caused by fungi, PR-10, when induced, acted with RNase function (Xie et al., 2010), degrading the RNA of the invading fungus and binding to cytokinin to mitigate the damage that the fungal stress caused (Agarwal et al., 2016). PR-10 expression has also been identified as triggering an HR response or reactive oxygen species (ROS) accumulation, leading to cell death (**Figure 5**) (Coram et al., 2008; Barreto et al., 2018). Some molecules influenced the susceptibility of the plant to the fungal pathogen. JAZ2 (Jasmonate-ZIM-domain) impairs JA sensitivity by decreasing the level of expression of JA response genes and increasing plant susceptibility (He et al., 2018). This protein interacts with PR-10. MPK15 also acted upstream of the JA and SA hormone signaling pathways, down-regulating them and leading to PR-10 expression decline, an effect that MAPK5 also causes (Xiong and Yang, 2003; Hong et al., 2019). The EP1 effector was identified forming a complex with PR-10, which led to the inhibition of callose deposition, compromising PR-10 mediated resistance (**Figure 5**) (Wang et al., 2021). Studies that observed the post-transcriptional regulation indicated that the DCL1a (Dicer-Like Ribonuclease) protein was induced by fungus causing disturbances in the miRNAome inhibiting the expression of PR-10 (Salvador-Guirao et al., 2018). miR395 and miR156a, for example, down-regulated WRKY26 and WRKYN1, respectively, leading to a decrease in PR-10 expression (Zhang et al., 2017).

#### 3.5.2 Bacteria

Among the articles, 24.53% investigated PR-10 in biotic stress caused by bacteria. Based on these studies, a biological model of the responses that were triggered in the plant cell involving PR-10 was designed (**Figure 6**). SA and JA positively activate PR-10 expression, either directly or by activating CDPK1 (Calcium-Dependent Protein Kinases) (He et al., 2018). PGIP1 (Polygalacturonase-Inhibiting Proteins) seemed to act by inducing the JA pathway, which consequently positively regulated PR-10 (Wu et al., 2019). In contrast, MPK15 and MAPK5 (Mitogen-Activated Protein Kinase) acted by negative regulation of the JA and SA pathway and PR-10, respectively (Xiong and Yang, 2003; Hong et al., 2019). In this signaling network, the JMJ705 (Jumonji C Domain) protein was involved in the up-regulation by methyl jasmonate. This demethylase removed the histone H3K27me3 methylation, activating PR-10 gene expression (**Figure 6**) (Li et al., 2013). Other molecules identified as regulating PR-10 were TFs, such as bZIP81.1, bZIP81.2 (Liu et al., 2019), WRKY62, WRKY76 (Liu et al., 2016), WRKY28, WRKY71 (Peng et al., 2010), WRKY11 (Lee et al., 2018), and WRKY67 (Vo et al., 2017). With the accumulation of PR-10 in the cell, its functions were induced, which included RNase activity, restauration of cellular homeostasis, or binding to molecules that participate in the SAR induction (**Figure 6**) (Carella et al., 2016; Huang et al., 2016). Studies have also highlighted that under conditions of biotic stress due to bacteria, an unknown kinase can phosphorylate PR-10 and form the PR-10/LRR1 complex (Leucine-rich repeat protein1). This complex enhanced PR-10 RNase activity, which led to increased production of ROS and promoted callose deposition, leading to cell death similar to the HR. These events can contribute to resistance to the stressor agent and consequently to the corresponding disease (Choi et al., 2012).

#### 3.5.3 Oomycetes

Interactions with oomycetes constituted 7.87% of the selected studies in the SR. Based on the systematization of these studies, the PR-10 signaling model in biotic stress caused by oomycetes was designed (**Figure 7**). The data indicated that the signaling cascade started with high levels of hormones SA, JA and ethylene (ET) that induced the expression of PR-10, as well as the TF ERF113 (Zhao et al., 2017; Goyal et al., 2021). The accumulation of PR-10 allowed the execution of its RNase function (**Figure 7**). In contrast, zeatin, a hormone from the cytokinin group, significantly inhibited RNase function (Fan et al., 2015). In works evaluating stress in plants due to oomycetes, a protein interaction between PR-10 and VDAC3 (Voltage-dependent, anion-selective channel) was identified. This complex induced cell death associated with ROS accumulation (**Figure 7**) (Ma et al., 2018). Finally, when analyzing the overexpression of PR-10, a change in the cell wall’s composition was observed, which could contribute to plant resistance (Castro et al., 2016). However, under stress by oomycetes, the grapevine (*Vitis vinifera*) overexpressing the PUB protein (Stress-Responsive U-box Protein) acted by regulating the expression of several PR-10 negatively or positively, depending on the PR-10 evaluated (Jiao et al., 2017).

#### 3.5.4 Insects

Plant-insect interactions accounted for 6.94% of the articles of this SR. In infections caused by insects, the SA signaling pathway activated PR-10 expression and the MAPK signaling pathway, after being triggered by PRR activation (**Figure 8**) (Melo-Braga et al., 2012). CC and NB domains of the resistance protein HBH14 (Brown Planthopper Resistance 14) were identified as participating upstream in the SA regulatory pathway and triggering some defense responses with increased generation of ROS, callose deposition and consequently, the up-regulation of PR-10 (Hu et al., 2017). The protein HLH61 (Basic Helix-Loop-Helix Protein 61) was also identified in this signaling cascade, up-regulating PR-10, while bHLH96 (Basic Helix-Loop-Helix Protein 96) repressed its expression. These molecules can interact forming a heterodimer. In addition to the positive and negative regulation of PR-10 these molecules caused, when plants were subjected to stress by insects, the hormonal pathways that regulated them were also antagonistic (**Figure 8**). The systematized data indicated that while JA and cis-12-oxo-phytodienoic acid (OPDA) induce HLH61, SA repressed it, as this happened with bHLH96. The JA pathway repressor JAZ3 also participated in this pathway; JAZ3 has been identified as interacting with bHLH96. A crosstalk seems to take place in two situations: the hormonal pathways for regulating the proteins of the HLH61/bHLH96 complex and the regulation of PR-10 that this complex mediates. This event may mediate plant resistance against the pathogen (**Figure 8**) (Wang et al., 2019).

#### 3.5.5 Nematodes

Interactions with nematodes constituted 4.62% of the selected and systematized articles. PR-10 was part of the SA and JA hormone signaling pathway (**Figure 9**). These hormones activated the expression of TFs MYB and WRKY, in addition to Non-expressor of pathogenesis-related genes1 (NPR1), which interacted with TGA TF and was a key mediator for inducing SAR, inducing the expression of PR-10. PR-10 expression favored forming a complex with SHMT08 (Serine Hydroxymethyltransferase) and SNAP18 (Soluble NSF Attachment Protein) (Lakhssassi et al., 2020). MO237 (Meloidogyne Graminicola Effector Protein) interacted with PR-10, suppressing the defenses of rice plants (*O. sativa*) that promote the nematode parasitism, as highlighted in these studies (Chen et al., 2018).

#### 3.5.6 Virus

Only 3.24% of the selected studies in the SR related to viral invasion. In all of them, PR-10 was activated in response to infection by viral agents. In virus infection, PR-10, and other highly expressed PRs, appeared to participate in SA signaling-induced SAR (**Figure 10**) (Kundu et al., 2019). CIPK30, a protein kinase that interacted with CBL (Calcineurin B-like proteins), positively regulated the induction of PR-10 (Liu et al., 2017). One study identified the P31 protein encoded by the RNA4 of the Beet Necrotic Yellow Vein Virus (BNYVV), which acted by specifically regulating PR-10, but only indirectly because there was no direct interaction between these molecules, leading to the appearance of severe symptoms (Wu et al., 2014). Cucumber mosaic virus (CMV) satellite RNA (satRNA) alleviates the symptoms CMV causes, inducing symptoms such as leaf epinasty and systemic necrosis involving programmed cell death (PCD), in addition to being related to the activation of transcription of several PR genes, including PR-10 (**Figure 10**). According to studies with tomato (*Lycopersicon esculentum*) disturbed by viruses, satRNA may be indirectly regulating PR-10 expression and, together with other molecules of the host defense system, triggered symptoms that led to the systemic necrosis of the plant (Xu et al., 2003) (**Figure 10**).

## 4 Discussion

### 4.1 Metadata reveal great interest in studies with PR-10 in biotic stress

The PR-10 family is involved in plant defense against biotic stresses. This evidence came from several studies that showed the improvement of plant resistance when PR-10 was overexpressed, when its induction was observed, or when its accumulation around sites that various stressors invaded, including viruses (Pühringer et al., 2000; Park et al., 2004; Wu et al., 2014), bacteria (Robert et al., 2001; Ahmed et al., 2012; Huang et al., 2016), fungi (Somssich et al., 1986; Pinto et al., 1995; Liu et al., 2004; Coram et al., 2008; Wu et al., 2016; Ribeiro et al., 2021), oomycetes (Meyer et al., 2016; dos Santos et al., 2020), nematodes (Kong et al., 2015; Hatzade et al., 2020), and insects (Wang et al., 2017; Coppola et al., 2019). Much knowledge about the role of PR-10 in biotic stress has been accumulated. In this sense, this SR was of great importance in compiling and systematizing existing data on the subject.

Regarding species studied, grapes, rice, corn, and wheat were the crops that most appeared in the studies that met the SR’s objectives (**Table S3**). All these data showed a trend, highlighting China as the main collaborating country in studies with an emphasis on PR-10 in biotic stress, with cereal as the investigative high spot. This corroborated current production data for these crops. Currently, China occupies the first position in the world production of rice and wheat and the second place in the production of corn, while the USA occupies the first place for this culture (FAO, Food and Agriculture Organization of the United Nations, 2022). Therefore, these cereals are of great economic importance worldwide and are periodically disturbed by stressors such as *Magnaporthe oryzae*, *Xanthomonas oryzae* in rice, *Rhizoctonia cerealis* in wheat, *Aspergilus flavus*, *Fusarium graminearum* in corn and grapevines, and *Plasmopara vitícola*. This aspect directly affects these crops’ production in the aforementioned countries, justifying the volume of studies published on this subject (Logrieco et al., 2002; Logrieco et al., 2003; Niño-Liu et al., 2006; Gessler et al., 2011; Hamada et al., 2011; Armijo et al., 2016; Sharma et al., 2021).

Often, research aimed at investigating the molecular mechanisms involving resistance and susceptibility of rice, corn, wheat and grapes in the interaction with the main pathogens of these crops has identified PR-10 as a defense molecule (Huffaker et al., 2011; Monteiro et al., 2013; Rong et al., 2016; Jain et al., 2017; Musungu et al., 2020). The groups of pathogens highlighted in this SR were fungi, bacteria and oomycetes, corroborating the data found in the differential expression analysis of PR-10 both at the transcript and the protein levels (**Figures 4A, B**). Studies with plants in interaction, mainly with bacteria and fungi, showed an up-regulation/induction of PR-10, indicating the importance of these molecules in studies involving plant resistance and the elucidation of their functions in this scenario.

### 4.2 Combined methodological tools can elucidate PR-10 action mechanisms

Techniques for investigating differential PR-10 expression levels under biotic stress identified in the SR included transcriptomics, RNA sequencing, small RNAs, cDNA libraries, microarray hybridization and RT-qPCR and proteomic techniques exploiting LC-MS/MS or MALDI-TOF MS systems. Together, studies using these tools totaled 95.8% of the amount selected for this SR (**Figure 3B**; **Table S4**).

Despite having been explored in only 9.16% of the studies, proteomics is a technique of great importance because it reveals changes at the translational and post-translational levels and has a more informative potential than measurement at the transcript level (Rampitsch and Bykova, 2012). Both methodologies complement each other. In studies combining both methodologies, whether what was being transcribed was being translated could be observed (Garavaglia et al., 2010). Together, these tools allowed a systematic investigation of the differential behavior of PR-10 expression, showing that high expression patterns were predominant, both at transcriptional and protein levels. Furthermore, it was possible to identify molecules that acted in the regulation of PR-10 at the transcriptional, post-transcriptional, and post-translational levels and in triggering resistance or susceptibility in the host subjected to biotic stress (**Table 3**; **Figures 5**–**10**).

Epigenetic studies did not stand out in the sample of articles, but they added relevant information about PR-10. JMJ705, which encodes a histone lysine demethylase, seems to remove the methylation present in a histone H3 that has the role of silencing genes involved in the response to stress in plants, including PR-10. Increased expression of JMJ705 caused depression of this gene (Li et al., 2013). Epigenetic studies in plants are important for the characterization of gene expression patterns (Iwasaki and Paszkowski, 2014). Transcriptional reprogramming by chromatin remodeling, during the plant’s immune responses, has already been described and it has brought together several TFs and genes with a defense role in plants (Alvarez et al., 2010). Deeper epigenetic studies with target genes during pathogen infection could provide further insights into the transcriptional behavior of PR-10 under biotic stress.

A single study from this SR exclusively applied *in silico* techniques to build co-expression networks (Jimmy and Babu, 2019). Despite this, a significant portion of the research identified here used *in silico* techniques combined with *in vitro* analysis, such as studies with transcriptomics and proteomics (Aritua et al., 2013; Coppola et al., 2019; Florencio-Ortiz et al., 2021).

In 23.61% of the studies, silencing or overexpression of PR-10 and other molecules that regulate or interact with PR-10 was applied. In addition to the functional characterization of PR-10 as a molecule active in the defense against pathogens of different types (Choi et al., 2012; He et al., 2013; Wu et al., 2016), this methodological strategy allowed identifying TFs (Peng et al., 2010; Liu et al., 2012; Zhao et al., 2017; Wang et al., 2019), PRRs (Xu et al., 2017), microRNAs (Devers et al., 2011), demethylases (Li et al., 2013) and proteins that played key roles in the molecular network triggered by the host under biotic stress (Harkenrider et al., 2016; Hong et al., 2019). All this information contributed to elucidating the defense mechanisms that involved PR-10 because they allowed access to information from the process of recognizing the pathogen to regulation at the transcriptional and post-transcriptional levels.

Techniques focusing on characterizing and investigating the functions PR-10 perform (8.8%) have shown its involvement in the accumulation of ROS, cell death (Castro et al., 2016), its subcellular localization (Ma et al., 2018), antifungal, antimicrobial action and activity of RNase and DNase (Xie et al., 2010; Wang et al., 2014; Fan et al., 2015) (**Table S3**). This low percentage of studies reveals that, although many studies related PR-10 to important roles in defense against pathogens, few carry out a deeper investigation, showing the relevance of this for more precise searches on the mechanism of action of this protein in biotic stress.

Methodological tools used together in different studies allowed advances in elucidating the behavior and roles PR-10 plays in plants subjected to biotic stressors through systematization (**Tables S4**; **S5**; **Figures 5**–**10**). However, they were represented in a very small sample compared to the number of studies selected in the SR, despite having significantly contributed to understanding the action of PR-10 in plant defense (**Table S5**) (Choi et al., 2012; Huang et al., 2016; Liu et al., 2019).

For example, one study used a combination of several analyses such as differential expression levels of transcripts, PR-10 overexpression, protein/protein interaction analysis, subcellular localization, and evaluation of plant resistance to fungal infection. This set of investigations revealed a multimeric complex between PR-10 proteins in the plant cell’s nucleus and cytoplasm, acting in the development and defense mechanisms of plants against fungi and bacteria (Lee et al., 2012).

The exploration of several other available tools, in combination, can provide more in-depth answers that contribute more precisely to elucidating the functional roles of PR-10 in biotic stress.

### 4.3 PR-10 transcriptional and translational overview

The articles that evaluated the differential levels of PR-10 selected for this SR revealed that, for the most part, this molecule was overexpressed under different stress conditions and at different evaluated times, regarding both transcripts and proteins (**Figure 4**; **Table S3**). However, the sample with studies on differential protein accumulation was quantitatively lower than the studies with transcription analysis.

A significant number of studies have identified that PR-10 is up-regulated in resistant varieties, both at the transcriptional and at the protein levels. Furthermore, inducing transcripts and proteins was higher and occurred earlier in resistant varieties than in susceptible ones (Polesani et al., 2010; Calla et al., 2014; Wu et al., 2019; Książkiewicz et al., 2021) (**Figure 4**; **Tables S1**; **S2**).

PR-10 proteins, in collaboration with other factors, participated in resistance against pathogens (Sharma et al., 2021). A significant majority of PR-10 studies have shown that its overexpression led to increased disease resistance (Xie et al., 2010; Fan et al., 2015; Castro et al., 2016). However, the mechanisms by which PR-10 helps in this process are still not well understood. Some speculate that RNase activity is important in this process through degrading the pathogen’s RNA, preventing the invader from growing (Park et al., 2004). Dual β-1,3-glucanase and RNase activity of a banana PR-10 was reported against *Aspergillus fumigatus* (Rajendram et al., 2022). Other speculations suggest providing resistance by activating hormonal signaling pathways (Thulke and Conrath, 1998; Hashimoto et al., 2004).

When induced, PR proteins were involved in HR and SAR responses (Choi et al., 2012; Jain and Kumar, 2015; Carella et al., 2016). SAR is a broad-spectrum plant defense response and long duration that leads to resistance to pathogens (Carella et al., 2016). HR involves PCD, acting as a signal to the plant, rather than a direct defense mechanism. ROS accumulation and PR-10 expression acted in coordination for HR induction (Choi et al., 2012). Thus, these are other proposed pathways for the induction of resistance by PR-10.

Few studies reported PR-10 down-regulation, which depended on the time and biotic stress assessed (**Figure 4A**; **Table S3**). PR-10 repression was caused by endophytic and mycorrhizal agents, which form mutualistic symbiotic associations with plants (Johnson et al., 2003; Grunwald et al., 2009; Mishra et al., 2018; Kiani et al., 2021), and when more than one PR-10 was evaluated in different genotypes or plant tissues (Ding et al., 2015; Abbasi et al., 2020; Irigoyen et al., 2020).

In strawberry (*Fragaria* × *ananassa*), in the interaction with the pathogen *Verticillium dahliae*, a differential behavior of the expression was seen, depending on the evaluated tissue. Four PR-10s were up-regulated in leaves, while eight were up-regulated in the root and five were positively expressed in both tissues. Two isoforms were induced *via* infection and two other identified members were not induced, showing these genes’ high variation of behaviors in biotic stress (Besbes et al., 2019).

PR-10 proteins are part of a large family with members that have described multifunctions and can be regulated in different ways, being widely found in several plant species (Sinha et al., 2020). Some functions that PR-10 performs include binding various hormone molecules, secondary metabolites, and having ribonuclease and defense activity against biotic and abiotic stressors (Marković-Housley et al., 2003; Fernandes et al., 2008; Xie et al., 2010; Sinha et al., 2020). It has also been shown that PR-10 genes were constitutively expressed in several plant tissues participating in their growth and development (Bantignies et al., 2000; Biesiadka et al., 2002). Thus, detecting PR-10 was possible even if the plant was not interacting with a stressor agent.

Symbiotic interactions between yellow lupine (*Lupinus luteus*) and *Bradyrhizobium* sp. led to the repression in mature root nodules of two PR-10, which are constitutively expressed in roots, suggesting that the plant’s defense mechanisms might be suppressed for symbiont recognition to be possible (Sikorski et al., 1999). The symbiosis between *Lolium arundinaceum* and *Neotyphodium* spp. caused the suppression of PR-10, which seemed necessary for establishing and developing the symbiotic relationship. PR-10 may also be expressed when regulating plant development rather than defense because its repression was easily overcome by infection with a foliar pathogen (Johnson et al., 2003).

PR-10 down-regulation also occurred in susceptible varieties shortly after being up-regulated (Mahomed and van den Berg, 2011; Giovannetti et al., 2015; Kumar et al., 2016; Chun and Chandrasekaran, 2019) and in some resistant varieties (McNeil et al., 2001; Fondevilla et al., 2011; Zhang et al., 2015; Fass et al., 2020). A study with tomato in the interaction with *Botrytis cinerea* showed that the SA signaling pathway promoted the development of the disease through NPR1, suppressing the expression of two defense genes dependent on JA. These data showed that the necrotrophic pathogen manipulates the SA signaling pathway, impacting the expression of defense genes (Rahman et al., 2012). In parallel, other studies indicated that the SA and JA signaling pathways acted in regulating PR gene expression (Choi et al., 2012; Rahman et al., 2012; Wu et al., 2016; Wang et al., 2019). The same regulation through the interaction between hormonal signaling pathways may occur in varieties that had PR-10 down-regulated.

Another important fact occurred in resistant varieties of hops, where systemic signaling induced PR proteins at high levels in the early stages of the disease, and then there was a decline in their expression, suggesting that the plant defense built up as the pathogen progresses, leading to an intense plant defense response by eliminating the fungus (Cregeen et al., 2015). In resistant varieties, the accumulation and subsequent decrease in PR-10 levels may be responsible for delaying the appearance of symptoms and for favoring the inhibition of spreading the pathogen.

Some studies that silenced or overexpressed molecules that regulated the plant’s resistance against stressor agents showed alterations in PR-10 expression levels, up or down. An example is the down-regulation of PR-10 in plants that silenced TF WRKY13 (Wang et al., 2019). In plants overexpressing TF *Ta*PIE1, PR-10 was up-regulated (Zhu et al., 2014). Plants that overexpressed or silenced the TF *Ta*GATA1 led to up- and down-regulation of PR-10, respectively (Liu et al., 2019). These studies contribute significantly to understanding PR-10 regulation and its roles in acquiring resistance by the plant.

A PR-10 of the MLP (major latex protein) type was also identified, negatively regulating plant defense, and two other MLPs that were repressed in plants subjected to biotic stressors (Wang et al., 2017; He et al., 2020; Pérez-Torres et al., 2021). Two fungal pathogens inhibited MLP expression. Its overexpression showed greater susceptibility to *Botryosphaeria berengeriana* f. sp. *Piricola* and *Alternaria alternata*, in addition to having weakened the hormonal pathways of signaling and having reinforced the cell wall, inhibiting the expression of TFs and other genes related to the pathogenesis (He et al., 2020).

Differential expression studies are often designed to analyze the response of genes under contrasting conditions. Molecules analyzed at a given time, tissue and condition, reflect the molecular dynamics underlying some type of stress, for example, functioning as an important tool in diagnosing and determining a metabolic, pathological or gene activation phenotype (Finotello and Di Camillo, 2015). These analyses contributed to selecting candidate genes that can facilitate the development of resistance in cultivated plants subjected to biotic stress, reducing the risk of loss. In addition, they can collaborate in the investigation of the roles and mechanisms of action of specific molecules (Xie et al., 2010; He et al., 2018; Wang et al., 2019).

The systematization of PR-10 differential expression data at the RNA and protein levels revealed that PR-10 plays an essential role in the plant’s defense against stressors. Furthermore, it may be a key molecule in resistant plants and a potential target for disease management.

### 4.4 Molecular interactions regulated the complex mechanism of action of PR-10 in plant defense

Plants respond to infection by pathogens of various types, from viruses to insects, using a very complex defense network that involves a signaling cascade in which several molecules participate (Kunkel and Brooks, 2002). Based on eligible studies in the SR, PR-10 may be involved in an intricate regulatory network and interactions to promote plant defense against bacteria, fungi, oomycetes, insects, nematodes, and viruses (**Figures 5**–**10**). These networks include phytohormones, TFs, microRNAs, demethylases, effectors, PRRs, PAMPs, MAPK signaling pathway molecules, proteins with roles in plant defense, and ROS.

The primary plant defense response begins with recognizing the pathogen by PRRs present on the plant cell’s surface. Chitin, for example, is a fungal cell wall component that is recognized by PRRs present on the plant cell’s surface, leading to pathogen-triggered immunity (PTI), which includes activating PR genes and ROS production (Jones and Dangl, 2006; Kaku et al., 2006; Meena et al., 2019). In the model of the plant defense response involving PR-10 in the interaction with fungi proposed in this SR (**Figure 5**), it was possible to identify a study with the characterization of three PRRs (Lyp1, Lyk7 and LysMe3) located in the plasmatic membrane of cotton. Silencing these PRRs led to a decrease in the synthesis of JA, SA and ROS; in addition, the activation of four PR defense genes, including PR-10 was impaired, as well as resistance to the fungal pathogen. PRRs were involved in activating processes to increase resistance to biotic stress caused by fungi downstream of hormone signaling pathways and PR protein synthesis, including PR-10 (Xu et al., 2017).

Effectors are molecules pathogens secreted to inhibit PTI and the plant cells’ receptors, called R proteins, recognize them (Stulemeijer and Joosten, 2008). Some effectors have been characterized as interacting with PR proteins during a pathogen attack, such as ToxA, Tox3 and CSEP0055 (Breen et al., 2016; Zhang et al., 2012; Lu et al., 2014). The specific interaction between PR-10 and the effector of the fungus *Blumeria graminis*, CSEP0064/BEC1054 that binds RNA, may influence the functionality of plant defense proteins and interfere with the RNase function performed by PR-10 in barley (Pennington et al., 2019). In the survey carried out in this SR, a PR-10 was identified that has its expression induced by ToxA and ToxB. However, the study did not portray the interaction between these molecules (Pandelova et al., 2012). Two studies involving the interaction of plants with nematodes and fungi (**Figures 9**; **5**), identified the MO237 and EP1 effectors, respectively, physically interacting with PR-10.

In fungi, the PR-10/EP1 complex led to inhibiting callose deposition (**Figure 5**) (Wang et al., 2021); in the interaction with nematodes, the PR-10/MO237 complex suppressed plant defense (**Figure 9**). MO237 also interacts with a 1,3-β-glucan synthase, a protein that acts in callose synthesis, promoting nematode parasitism (Verma and Hong, 2001; Chen et al., 2018). Because callose deposition is a response related to the plant’s immune system to resist stress (Luna et al., 2011; Dumanović et al., 2021) inhibiting this response led to suppress plant defense and disease susceptibility (Chen et al., 2018; Wang et al., 2021). It is important to emphasize that, with the exception of the models proposed in this SR for viruses and nematodes (**Figures 9**; **10**), all others involved callose deposition, and in bacteria, this event was associated with cell death similar to the HR (**Figure 1**).

In the survey conducted in the SR, cell death or SAR was in all biological models involving PR-10, with the exception of the interaction with insects. HR and SAR on interaction with bacteria, HR on fungi, cell death on oomycetes, SAR on nematodes, and SAR and PCD on interaction with viruses (**Figures 5**–**10**). A study selected from the SR showed that the overexpression of the PR-10/LRR1 complex conferred greater resistance of *Arabidopsis thaliana* against infection by the bacteria *Pseudomonas syringae* pv tomato and by the oomycete *Hyaloperonospora arabidopsidis*. The complex these proteins formed triggers an increased ROS, callose deposition, and enhanced PR-10 phosphorylation to perform its RNase function. LRR1 physically interacted with PR-10, up-regulating cell death that resembled HR triggered by PR-10, and signaling defense against *P. syringae* infection (Choi et al., 2012). An MLP type PR-10 has also been linked to SAR induction through its activity in binding and transporting abundant molecules in the plant’s phloem (Carella et al., 2016).

HR is a form of PCD and is classically associated with localized necrosis of plant cells in the regions and early times when the pathogen is trying to infect the plant. It is not known for sure whether HR is a cause or consequence of pathogen death (Hammond-Kosack and Jones, 1996; Balint-Kurti, 2019). HR can induce SAR, causing broad-spectrum systemic resistance. SAR develops further as a result of the activation of effector-triggered immunity (ETI) and PTI, resulting in resistance against the stressor agent because it acts by sending a signal that circulates over long distances in the plant, reaching regions that were not damaged to induce defense mechanisms (Fraser, 1982; Vallad and Goodman, 2004). SAR requirements involve the accumulation of SA and PR proteins (Fraser, 1982; Vallad and Goodman, 2004).

Depending on the model of the biological network proposed in this SR, for bacteria, fungi, oomycetes, nematodes, insects and viruses (**Figures 5**–**10**), a different behavior of the hormonal pathways occurred. These pathways led to PR protein recruitment (Xu et al., 2017). The hormones SA, JA and ET were included in the group of molecules that the pathogens induced and they play the main role in stimulating resistance to pathogenic stressors agents (Robert-Seilaniantz et al., 2011). This accumulation activated signaling pathways that involved inducing the expression of several genes, acting as protagonists in the signaling networks involved in the defense response in plants. The interaction and crosstalk between these hormonal pathways allowed the plant’s inducible defense system to adapt to different types of invaders. This data demonstrated why the hormonal pathways behaved in different ways.

In the selection of studies related to the interaction of plants with oomycetes, one showed the induction of PR-10 by ET, SA and JA (Fan et al., 2015). The hormone OPDA has also been characterized in the up-regulation of HLH61 protein that interacted with bHLH96, mediating plant defense through the regulation of PR genes, including PR-10 and mediating crosstalk involving SA and JA (Wang et al., 2019).

NPR1, for example, is a molecule expressed downstream of the SA signaling pathway, which was involved in the crosstalk involving SA and JA (Spoel et al., 2003; Pieterse and Vanloon, 2004). Mutant Arabidopsis plants for this gene had the SA pathway impaired and showed that the antagonistic effect of SA on JA signaling required the NPR1 protein (Spoel et al., 2003). NPR1 also acted as a key regulator for triggering SAR and in transducing signals for activating PR gene expression. In the model proposed for the interaction of plants with nematodes in this SR, an NPR1 acted indirectly in activating PR-10. The TF WRKY13 promoted the activation of NPR1, which interacted with TGA TFs, leading to activating the transcription of PR genes including PR-10 (**Figure 9**) (Kumar et al., 2016).

In this systematization, the TFs WRKY, AP2/ERF, bZIP, RAV2, GATA1 and MYB also acted in regulating PR-10 expression in the proposed biotic stresses (**Table 3**; **Figures 5**–**10**). A rice TF *Os*bZIP acts by positively regulating JA levels, in addition to interacting with PR-10 (Liu et al., 2019). Wheat *Ta*GATA1 activate PR-10 in the defense against *Rhizoctonia cerealis* and they are related to cytokinin or JA stimulus (Liu et al., 2019). *Ta*PIMP2, a TF MYB from wheat, positively modulated the expression of PR genes, including PR-10, contributing to resistance to *Bipolaris sorokiniana* (Wei et al., 2017). *Gm*ERF113, a soybean TF AP2/ERF, up-regulated the expression of PR-1 and PR-10 genes, improving resistance of susceptible varieties to *Phytophthora sojae* (Zhao et al., 2017). WRKY26, WRKYN1 from *Malus* × *domestica*, were related to PR-10 activation conferring apple resistance to infection by *Alternaria* f. sp. *mali* (Zhang et al., 2017). Other WRKY TFs, such as WRKY11 (Lee et al., 2018), WRKY13 (Wang et al., 2019), WRKY2 (Dabi et al., 2020), WRKY67 (Vo et al., 2017), WRKY62, WRKY28, WRKY71, and WRKY76 (Peng et al., 2010) were identified in this SR and acted by regulating PR-10 expression in biotic stress. Taken together, these data revealed the regulatory network that PR-10 is part of, involving phytohormones and TFs, to help guide the plant’s resistance to the stressor agent.

Post-transcriptional regulation is also one of the pathways through which indirect regulation of PR-10 in biotic stress has been identified. The increase in Md-miR395 and Md-miR156ab from *Malus* × *domestica* led to a decrease in the transcription of TFs WRKY26 and WRKYN1, respectively, which, in turn, led to the down-regulation of PR-10 expression, resulting in susceptibility to *A. alternaria* f. sp. *mali* (Zhang et al., 2017). The susceptibility caused by the decrease of WRKY TFs that regulate PR-10 reveals the importance of this molecule for maintaining the plant defense signaling cascade. A sequencing analysis of degradome from mycorrhizal roots of *Medicago truncatula* also detected PR-10 as a target of miR1510b* (Devers et al., 2011). The cleavage of important genes, such as PR-10, by miRNAs, demonstrate the great importance of these regulatory pathways for establishing symbiosis.

In the proposed models of signaling networks in response to bacteria (**Figure 6**), fungi (**Figure 5**) and insects (**Figure 8**), mitogen-activated kinases (MAPK) appear to participate in regulating PR-10, even if indirectly (Xiong and Yang, 2003; Melo-Braga et al., 2012; Hong et al., 2019). MAPK was activated in response to plant recognition of pathogen-derived signals (Zhang and Klessig, 2001). In a study of bacteria and fungi interacting with plants, overexpression of OsMPK15 down-regulated the expression of PR genes, including a PR-10 and genes related to oxidative stress. In contrast, the accumulation of SA and JA decreased in these strains. OsMPK15 down-regulated disease resistance by modulating SA and JA hormone pathways (**Figures 5**; **6**) (Hong et al., 2019). The plant response to bacteria also showed down-regulation of PR-10 by OsMAPK5 (**Figure 6**) (Xiong and Yang, 2003). In contrast, in the plant’s response to the interaction with insects, the MAPK pathway positively stimulated the expression of PR-10 (**Figure 8**) (Melo-Braga et al., 2012).

Finally, when expressed, PR-10 performed RNase and DNase functions, antifungal and antimicrobial action (Wang et al., 2014; Fan et al., 2015; Mena et al., 2020) and bound to molecules through the hydrophobic cavity present in these proteins (Carella et al., 2016). RNase and DNase activity often associated with defense strategies in plants (Park et al., 2004; Kim et al., 2008; Agarwal et al., 2013; He et al., 2013; Wang et al., 2014). PR-10 ribonuclease activity and mRNA accumulation corresponding to this molecule were detected in phosphate-deficient rice cells. This indicates that PR-10 may hydrolyse RNA to release Pi (phosphate) and nucleosides that will be transferred to metabolic pathways. Thus, PR-10 may be functionally related to phosphate recycling, promoting cellular homeostasis associated with resistance to pathogens (Huang et al., 2016).

A relationship between RNA hydrolysis and cytokinin binding has also been identified. This hormone significantly inhibited PR-10 RNase activity (Fan et al., 2015), probably because they bind to this molecule. These data suggested that PR-10 acted in conjunction with the signaling pathway involving cytokinin, causing greater tolerance to stress (Agarwal et al., 2016).

Often, PR-10 has been described as cytoplasmic proteins, suggesting that their functions are carried out in this cellular compartment. However, this SR revealed the location of PR-10 in the nucleus forming complexes with other PR-10 (Lee et al., 2012), in the apoplast in the PR-10/LRR1 complex that enhanced PR-10 phosphorylation and its RNase activity (Choi et al., 2012), in the mitochondria forming a complex with VDAC3 promoting cell death in the host (Ma et al., 2018) and in the cell membrane (Fan et al., 2015). These data suggested that PR-10 may interact with other proteins to perform functions in other subcellular compartments.

## 5 Conclusion

This review summarizes information from data produced and accumulated between the years 2003-2021 on the action of PR-10 in plants under biotic stress. PR-10 is markedly up-regulated/accumulated in the different predicted plant/pathogen interactions, with predominance in resistant varieties, and it can be seen as a potential marker of resistance in pathosystems. Essential molecules for the plant’s immune system and the control of gene expression are part of the complex biological network in which PR-10 is involved, such as TFs (WRKY, AP2/ERF, bZIP, RAV2, GATA1 and MYB), pathway hormones SA and JA, PRRs (Lyp1, Lyk7 and LysMe3), microRNas, and the JMJ705 demethylase. Two effector molecules from fungi and nematodes that formed complexes with PR-10 promoted the suppression of host immune responses. The specificity of these interactions and new proposals for the analysis of effector/PR-10 interaction can collaborate with a better understanding of the mode of action of PR-10 against different types of biotic stressors. The systematized results allow inferring about the lack of combined tools in studies for a deeper understanding of the modes of action of PR-10. The use of technologies that aim to characterize the interaction between molecules, subcellular location, epigenetic mechanisms and post-transcriptional events can contribute with more accurate information about these molecules’ mode of action. Furthermore, the RNase and DNase functions of PR-10 related to its antifungal and antimicrobial action need further investigation because they crucially contribute to the models of biological networks proposed in this SR. Although some research has been conducted to understand the mechanism of action of PR-10, here, we suggest some questions that still need clarification. What are the molecules forming complexes with PR-10 and what processes do these interactions trigger? Are the RNase and DNase functions performed by PR-10 related to its role in plant defense or in plant tissue growth and development? What can the interaction of PR-10 with effectors tell about the functional and behavioral specificity of this molecule in the face of each stressor? The SR allowed identifying aspects that contribute significantly to understanding the mechanisms of action of PR-10 and it revealed new perspectives of studies. Furthermore, the data collected in this SR suggest that PR-10 may act as a marker of plant resistance to biotic stress.

## Data availability statement

The original contributions presented in the study are included in the article/**Supplementary Material**. Further inquiries can be directed to the corresponding author.

## Author contributions

NSL, ASS, CPP and FM contributed to conception and design of the study. NSL, ASS and DPSN performed the planning and execution steps. NSL performed the summarization step, analyzed and organized the data. ASS and DPSN participated in figure design. NSL and FM wrote the first draft of the manuscript. FM advised NSL. All authors contributed to the article and approved the submitted version.
