# Supplementary material for: Pathogenesis-related protein 10 in resistance to biotic stress: progress in elucidating functions, regulation and modes of action
Source: Front Plant Sci. 2023 Jul 4;14:1193873. doi: 10.3389/fpls.2023.1193873 (PMC10352611; doi:10.3389/fpls.2023.1193873)
Supplement: Supplementary file 2 [file DataSheet_1.pdf]

PRISMA 2009 Checklist

Section/topic	#	Checklist item	Reported on page #
**TITLE**			
Title	1	The PR-10 protein in resistance to biotic stresses: progress in the elucidation of functions, regulation and modes of action.	Title page, Page 1
**ABSTRACT**			
Structured summary	2	Family 10 pathogenesis-related proteins (PR-10) are widely distributed in the plant kingdom. They are multifunctional proteins, constitutively expressed in all plant tissues, playing a role in growth and development or being induced in stress situations. Several studies have investigated the preponderant role of PR-10 in plant defense against biotic stresses, however, little is known about the mechanisms of action performed by these proteins. This is the first systematic review carried out in order to gather information on the subject and reveal the possible mechanisms of action performed by PR-10. Therefore, we searched three databases (PubMed, Web of Science and Scopus). In order to avoid bias, a protocol with inclusion and exclusion criteria was previously prepared. In total, 216 articles related to the proposed objective for this study were selected. The participation of PR-10 was revealed in the defense of the plant against several stressor agents such as virus, bacteria, fungi, oomycetes, nematodes and insects, and studies involving fungi and bacteria were predominant in the sample. Studies with combined techniques showed a compilation of relevant information about PR-10 in biotic stress that collaborate with the understanding of the mechanisms of action performed by this molecule. The upregulation of PR-10 was predominant under different conditions of biotic stress over time, in addition to being more expressive in resistant varieties both at the transcriptional level and at the protein level. Biological models have been proposed that reveal an intrinsic web of molecular interactions involving PR-10 modes of action. These include hormonal pathways, transcription factors, physical interactions with effector proteins or Pattern Recognition Receptors (PRRs) and other molecules involved with the plant's defense system. The molecular web involving PR-10 reveals how the plant's defense response is mediated, either to trigger susceptibility or, based on data systematized in this review, more frequently, plant resistance to the disease.	Abstract, Page 1
**INTRODUCTION**			
Rationale	3	The information gathered in this review contributes to the understanding of the still unresolved mechanism of action in which the pathogenesis-related protein of family 10 (PR-10) is involved.	Introduction, Page 2
Objectives	4	- Identify whether there are differences in the response of this protein in plant varieties resistant and susceptible to biotic stress;<br>- Identify functions and mechanisms of action of PR-10 that are related to its defense role against biotic stresses;<br>- Verify whether the applied methodologies have allowed elucidating the role of PR-10 proteins in biological processes.	Introduction, Page 4
**METHODS**			
Protocol and registration	5	The OR connector was used to group the synonymous keywords and AND to group the main parts. Thus, the search string used in all databases was (PR10 OR “PR 10” OR PR-10) AND (“biotic stress” OR “biotic stresses”). For this research, only articles in English, available in academic channels, were used.	Material and methods, Execution,

			Page 5 and Planning, Page 4
Eligibility criteria	6	Only experimental articles published in English were selected.	Material and methods, Execution: Selection and extraction, Page 5 and 6
Information sources	7	Searches were carried out in previously selected databases: Web of Science, Scopus and PubMed Central. The results were imported into BIBTEX formats, compatible with the StArt tool.	Material and methods, Page 5
Search	8	Search strings in selected databases were tested. The string was added to the database search tab and all files found were saved in BibTex format on the computer. Later, they were exported to Start.	Material and methods, Page 5
Study selection	9	The studies were selected from the databases based on the title, abstract and keywords containing the central theme of the review.	Material and Methods, Page 5
Data collection process	10	The analysis of the studies answered the following questions:<br>1- What were the plant species in which PR-10 was characterized?<br>2- What stressor biotic agents are portrayed in studies with PR-10?<br>3- Are the methodologies in studies with PR-10 effective to elucidate the mechanisms of action against biotic stresses?<br>4- Is there differential PR-10 expression in varieties susceptible or resistant to biotic stresses?<br>5- What are the functions of PR-10 in the defense against biotic stresses?<br>6- What are the mechanisms of action performed by the PR-10?	Material and methods, in table 1, Page 4 e 5
Data items	11	Species studied; Study countries; Stressor agent; Methodology used; Resistant variety; Susceptible varieties; Level of transcripts; Times evaluated in the analysis of transcripts; Protein accumulation; Times evaluated in protein analyses; Molecules that regulate PR-10 expression; Function performed; Mechanism of action.	Material and methods, Page 5
Risk of bias in individual studies	12	We followed the inclusion and exclusion criteria and also adopted the PICOS strategy and PRISMA guidelines.	Material and methods,

PRISMA 2009 Checklist

			Page 5
Summary measures	13	We did not employ meta-analyses in the study.	N/A
Synthesis of results	14	We did not employ meta-analyses in the study.	N/A

Section/topic	#	Checklist item	Reported on page #
Risk of bias across studies	15	We follow the pre-established criteria in the execution protocol.	Material and methods, Page 5
Additional analyses	16	No additional methods of analysis were used.	N/A
Study selection	17	A total of 579 articles returned from searches in the databases, 518, 28 and 33 from the respective databases, Pubmed, Web of Science and Scopus. Electronic searches in each database corresponded to 89.46%, 4.83% and 5.69% of studies in the respective databases, mentioned above. Although the same string was used in different databases, PuMed contributed more studies to the systematic review. In the first selection of articles in the StArt software, 329 articles were excluded because they were ineligible for the inclusion criteria of the review, with 279 being excluded based on reading the titles and abstracts, and 50 based on the complete reading of the articles. The automatic tool detected 34 duplicate articles. Therefore, a resulting sample of 216 articles was included in the systematic review, and these met at least one inclusion criterion.	Results, Page 6
Study characteristics	18	Research questions guided data extraction in all selected articles.	Results, Page 6-25
Risk of bias within studies	19	Seguimos os critérios de inclusão para todos os estudos selecionados.	Material and methods, Page 4 and 5
Results of individual studies	20	We followed the inclusion criteria for all selected studies.	Results, Page 6-25
Synthesis of results	21	We do not employ meta-analysis.	N/A
Risk of bias across studies	22	We do not perform a risk assessment.	N/A
Additional analysis	23	No additional methods of analysis were used.	N/A

DISCUSSION			
Summary of evidence	24	- The Metadata reveal great interest in studies with PR-10 in biotic stress: China appears as the main collaborating country in studies with emphasis on PR-10 in biotic stress.<br>- Grains such as rice, wheat and corn were the crops with investigative emphasis. This corroborates the current production data for these crops.<br>- The main stressors were *Magnaporthe oryzae* and *Xanthomonas oryzae* in rice, the necrotrophic fungus *Rhizoctonia cerealis* in wheat, the fungi *Aspergilus flavus* and *Fusarium graminearum* in maize and the oomycete *Plasmopara viticola* in grapevine.<br>- The methodologies applied in the studies were effective for the investigation of the role of PR-10 in biotic stress when they were combined with other methodologies.<br>- PR-10 is predominantly transcriptional and protein upregulated in plant interaction with different biotic stressors, different times and in contrasting varieties.<br>- PR-10 is regulated by several molecules that participate in the plant's defense against biotic stress, including transcription factors, effectors and PRRs.	Discussion, Page 26-40
Limitations	25	Despite the importance of understanding the role played by PR-10 in plant defense against biotic stressors, few studies have been carried out in depth and focused on elucidating the mechanisms of action of PR-10.	Discussion, Page 31
Conclusions	26	This review summarizes information from data produced and accumulated in recent years on the action of PR-10 in plants under biotic stress through systematization.<br>PR-10 is markedly up-regulated/accumulated in the different predicted plant/pathogen interactions, with predominance in resistant varieties, and can be seen as a potential marker of resistance in pathosystems.<br>Essential molecules for the plant's immune system and control of gene expression are part of the complex biological network in which PR-10 is involved, such as transcription factors (WRKY, AP2/ERF, bZIP, RAV2, GATA1 and MYB), pathway hormones SA and JA, PRRs (Lyp1, Lyk7 and LysMe3), in addition to microRNAs, and the JMJ705 demethylase.<br>Two effector molecules from fungi and nematodes that formed complexes with PR-10, promoted the suppression of host immune responses. The specificity of these interactions and new proposals for analysis of effector/PR-10 interaction can collaborate with a better understanding of the mode of action of PR-10, against different types of biotic stressors.<br>The systematized results allow inferring about the lack of combined tools in studies for a deeper understanding of the modes of action of PR-10. The use of technologies that aim to characterize the interaction between molecules, subcellular location, epigenetic mechanisms and post-transcriptional events can contribute with more accurate information about the mode of action of these molecules. Furthermore, the RNAse and DNAse functions of PR-10 related to its antifungal and antimicrobial action need further investigation, as they are crucial to contribute to the models of biological networks proposed in this systematic review.<br>Although in recent years, research has been carried out to understand the mechanism of action of PR-10, here we suggest some questions that still need to be clarified:<br>- *What other molecules form complexes with PR-10 and what processes are triggered by this interaction?*<br>- *Is the RNAse and DNAse function performed by PR-10 more related to its role in plant defense or to plant tissue growth and development?*	Conclusion, Page 41-42

		- What can the interaction of PR-10 with effectors reveal to us about the functional and behavioral specificity of this molecule, facing each stressor agent?<br><br>The Systematization of the literature allowed identifying aspects that contribute significantly to the understanding of the mechanisms of action of PR-10 and reveals new perspectives for studies on the subject. Furthermore, it demonstrates the potential of this molecule as a precursor target of plant resistance to biotic stress.	
**FUNDING**			
Funding	27	This work was carried out without any source of funding.	N/A

From: Moher D, Liberati A, Tetzlaff J, Altman DG, The PRISMA Group (2009). Preferred Reporting Items for Systematic Reviews and Meta-Analyses: The PRISMA Statement. PLoS Med 6(7): e1000097. doi:10.1371/journal.pmed1000097

For more information, visit: **www.prisma-statement.org**.

StArt - State of the Art through Systematic Review

**Title:**	The PR-10 protein in resistance to biotic stresses: progress in the elucidation of functions, regulation and modes of action.
**Researchers:**	Natasha dos Santos Lopes;<br>Ariana Silva Santos;<br>Diogo P. S. Novaes;<br>Carlos Priminho Pirovani;<br>Fabienne Micheli.
**Description:**	To carry out this systematic review, articles on the roles of protein related to the pathogenesis of family 10 with a focus on biotic stress were selected.
**Objective:**	- Identify whether there are differences in the response of this protein in plant varieties resistant and susceptible to biotic stress;<br>- Identify functions and mechanisms of action of PR-10 that are related to its defense role against biotic stresses;<br>- Verify whether the applied methodologies have allowed elucidating the role of PR-10 proteins in biological processes.
**Main questions:**	1- What were the plant species in which PR-10 was characterized?<br>2- What stressor biotic agents are portrayed in studies with PR-10?<br>3- Are the methodologies in studies with PR-10 effective to elucidate the mechanisms of action against biotic stresses?<br>4- Is there differential PR-10 expression in varieties susceptible or resistant to biotic stresses?<br>5- What are the functions of PR-10 in the defense against biotic stresses?<br>6- What are the mechanisms of action performed by the PR-10?
**Key words:** Stress; Mechanism; defense; Systematic review.	
**Search source selection criteria:** Scientific articles only.	
**Language of studies:** Only in English.	
**Research method:** Articles found in widely available scientific databases.	
**Search database:** Scopus; PubMed; Web Of Science.	
**Inclusion criteria:** Articles in English; Primary articles; Studies with biotic and abiotic stress together; Studies with PR-10 and biotic stress; Articles with the action of PR-10 in contact with a biotic stressor agent; Articles that are aligned with the objective of the systematic review.	

**Exclusion Criteria:** Articles that are not aligned with the objective of the systematic review; Studies with abiotic stress only; Review articles; Manuals; Technical reports; book chapters; Theses and dissertations; Abstracts; Articles published in event annals.
**Definition of types of studies:** Based on inclusion and exclusion criteria.
**Initial selection of studies:** Articles that contain in the title, abstract or keywords, the terms "PR10" or "PR" and "Biotic stress" or "Defense" or "Resistance".
**Evaluation of the quality of studies:** Articles that met one of the inclusion criteria and none of the exclusion criteria.
**Data Extraction Strategy:** Abstract (objective + conclusion); Key words; Species studied; Study countries; Stressor agent; Methodology used; Resistant variety; Susceptible varieties; Level of transcripts; Times evaluated in the analysis of transcripts; Protein accumulation; Times evaluated in protein analyses; Molecules that regulate PR-10 expression; Function performed; Mechanism of action.
**Data summary:** Graphs, tables and/or figures.
